# Cerium oxide nanoparticles in wound care: a review of mechanisms and therapeutic applications

**DOI:** 10.3389/fbioe.2024.1404651

**Published:** 2024-05-20

**Authors:** Shouying Chen, Yiren Wang, Shuilan Bao, Li Yao, Xiao Fu, Yang Yu, Hongbin Lyu, Haowen Pang, Shengmin Guo, Hongwei Zhang, Ping Zhou, Yun Zhou

**Affiliations:** ^1^ School of Nursing, Southwest Medical University, Luzhou, China; ^2^ Wound Healing Basic Research and Clinical Application Key Laboratory of Luzhou, School of Nursing, Luzhou, China; ^3^ Department of Pediatrics, West China Second Hospital, Sichuan University, West China School of Nursing, Sichuan University, Chengdu, China; ^4^ Key Laboratory of Birth Defects and Related Diseases of Women and Children, Ministry of Education, Chengdu, China; ^5^ School of Basic Medical Science, Southwest Medical University, Luzhou, China; ^6^ Department of Ophthalmology, The Affiliated Hospital of Southwest Medical University, Luzhou, China; ^7^ Department of Oncology, The Affiliated Hospital of Southwest Medical University, Luzhou, China; ^8^ Department of Nursing, The Affiliated Hospital of Southwest Medical University, Luzhou, China; ^9^ Department of Transfusion, The Affiliated Hospital of Southwest Medical University, Luzhou, China; ^10^ Department of Radiology, The Affiliated Hospital of Southwest Medical University, Luzhou, China; ^11^ Department of Psychiatric, The Zigong Affiliated Hospital of Southwest Medical University, Zigong, China; ^12^ Zigong Psychiatric Research Center, Zigong, China

**Keywords:** cerium oxide, wound healing, mechanism, composite materials, nanotechnology, wearable sensor, toxicity, interdisciplinary

## Abstract

Skin wound healing is a complex and tightly regulated process. The frequent occurrence and reoccurrence of acute and chronic wounds cause significant skin damage to patients and impose socioeconomic burdens. Therefore, there is an urgent requirement to promote interdisciplinary development in the fields of material science and medicine to investigate novel mechanisms for wound healing. Cerium oxide nanoparticles (CeO_2_ NPs) are a type of nanomaterials that possess distinct properties and have broad application prospects. They are recognized for their capabilities in enhancing wound closure, minimizing scarring, mitigating inflammation, and exerting antibacterial effects, which has led to their prominence in wound care research. In this paper, the distinctive physicochemical properties of CeO_2_ NPs and their most recent synthesis approaches are discussed. It further investigates the therapeutic mechanisms of CeO_2_ NPs in the process of wound healing. Following that, this review critically examines previous studies focusing on the effects of CeO_2_ NPs on wound healing. Finally, it suggests the potential application of cerium oxide as an innovative nanomaterial in diverse fields and discusses its prospects for future advancements.

## Graphical Abstract

**Figure:**
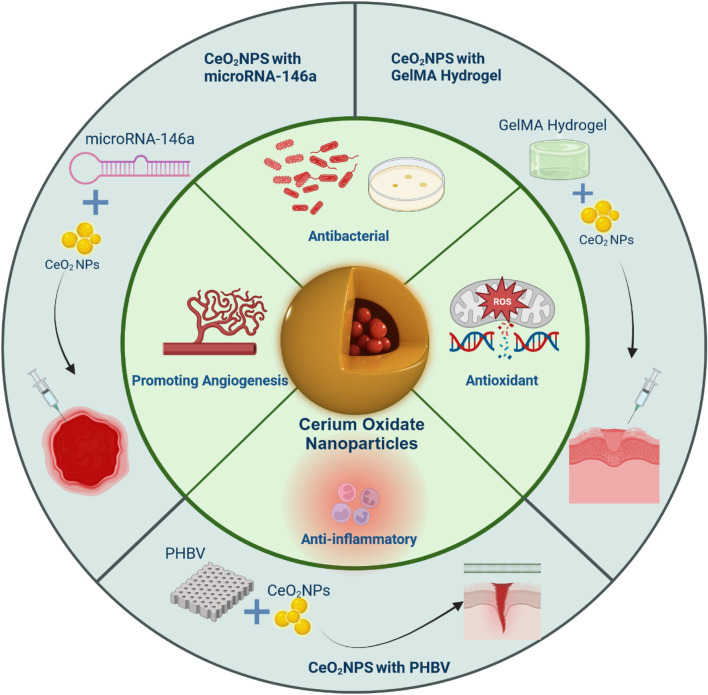
This article demonstrates the therapeutic mechanism of CeO_2_ NPs in wound healing. Then, the application of cerium oxide as a new nanomaterial in wound healing and the prospect of its future development are presented.

## 1 Introduction

Chronic and acute wounds pose significant health challenges globally. According to recent statistics from the World Health Organization, millions of people worldwide suffer from acute or chronic wounds each year resulting from accidents, trauma, or chronic diseases (Burgess et al., 2021). These wounds not only significantly impact individuals’ quality of life but also place a heavy burden on healthcare systems. It is estimated that global medical expenses related to wound care amount to tens of billions of dollars annually (Piipponen et al., 2020). Alarmingly, delays or deterioration in the wound healing process can lead to more severe complications such as infections, tissue necrosis, limb loss, and even life-threatening situations. Therefore, it is crucial to provide timely and effective wound care to protect wounds, promote healing, prevent infections and complications, and enhance patients’ comfort and psychological recovery.

Skin wound healing is a complex process that requires precise regulation of various cellular and molecular events. Numerous diseases can affect the different stages of wound healing. Consequently, discovering new therapeutic strategies that can enhance the healing of both acute and chronic wounds has always been a crucial area of research in the medical field. There is a wide range of treatments available for the healing of both acute and chronic wounds. Standard clinical treatments for wounds encompass various approaches such as skin perfusion recovery, infection management, metabolic control, complication treatment, local wound care, debridement, administration of growth factors, pressure regulation, hyperbaric oxygen therapy, and physical therapy (Comino-Sanz et al., 2021; Wang et al., 2023). Conventional dressings, such as gauze, are extensively utilized in clinics due to their convenience, affordability, and ability to quickly stop bleeding. However, they may hinder wound care and healing by promoting bacterial infections and insufficient presence of endogenous growth factors around the wound (Guo and DiPietro, 2010). Additionally, the prolonged treatment process can lead to secondary harm and have negative effects on the mental and physical wellbeing of patients. Therefore, further research on novel wound dressings that accelerate wound healing is necessary. In recent years, nanotechnology has become a promising field for developing innovative methods for wound healing due to advances in tissue engineering and regenerative medicine. The distinctive properties of nanomaterials, including their small size, high surface area-to-volume ratio, and customizable surface chemistry, make them promising candidates for wound healing applications. Nanotechnology-based methods have shown significant potential in improving wound healing outcomes through the promotion of cell proliferation and migration, inflammation reduction, angiogenesis induction, and broad-spectrum antibacterial activity. Various novel nanomaterials, including zinc oxide (Lin et al., 2017), silicon dioxide (Yang et al., 2022), gold and silver nanoparticles (Sharma et al., 2019), and cerium oxide, have been widely investigated for their potential in wound healing (Thakur et al., 2019). These nanomaterials have demonstrated positive effects on wound healing, such as excellent biocompatibility, re-epithelialization, antibacterial properties, and reduced scar formation (Baptista-Silva et al., 2021). Among them, CeO_2_ NPs are particularly noteworthy due to their outstanding antioxidant, anti-inflammatory, antibacterial, and angiogenic abilities, making them one of the most promising materials in this field.

CeO_2_ NPs exhibit advantageous physicochemical properties. CeO_2_ NPs have found extensive applications across diverse fields (Li et al., 2020), particularly in biomedicine, demonstrating potential for antioxidant therapy, cancer treatment, and bioimaging. Furthermore, owing to the thermal stability, favorable mechanical properties, exceptional oxygen storage capacity, and high retention rate of conjugated enzymes, the utilization of CeO_2_ NPs exhibits tremendous potential in wound healing (Lindholm and Searle, 2016; Singh et al., 2020).

Studies indicate that CeO_2_ NPs have the potential to positively impact biological processes, including inflammatory responses, cell proliferation, cell migration, and cell differentiation, thereby enhancing wound healing. Firstly, as potent antioxidants, CeO_2_ NPs can alleviate damage to wound tissue caused by inflammatory responses and oxidative stress (Hu et al., 2014). Secondly, they can modulate multiple cell signaling pathways, promoting cell proliferation and migration, thereby accelerating the wound healing process (Xu and Qu, 2014). Additionally, CeO_2_ NPs are believed to promote tissue regeneration and repair, further facilitating wound healing (Li et al., 2021; Sangha et al., 2024). These findings offer robust theoretical support for the utilization of CeO_2_ NPs in the realm of wound healing.

Although some promising results have been obtained, a comprehensive and in-depth understanding of the mechanisms of action and applications of CeO_2_ NPs in wound healing is still lacking. This review thus centers on the mechanisms of action of CeO_2_ NPs in wound healing and their potential applications. Through synthesizing existing research findings, this review aims to comprehensively elucidate the potential therapeutic mechanisms of CeO_2_ NPs in wound treatment and healing, offering a significant reference for future research and clinical applications.

## 2 The process and mechanism of wound healing

### 2.1 Hemostasis

Hemostasis is the first step in wound healing and aims to prevent bleeding. When the skin is damaged, platelets come into contact with collagen, leading to activation and aggregation, and the central thrombin triggers the formation of a fibrin mesh, which strengthens the platelet cohesive mass into a stable clot, effectively stopping bleeding (Velnar et al., 2009; Broughton et al., 2006). The most dominant feature of the hemostatic phase is the platelet, which is the main contributor to hemostasis and clotting. Platelet cytoplasm contains growth factors and cytokines such as platelet-derived growth factor (PDGF), transforming growth factor-β (TGF-β), epidermal growth factor and insulin-like growth factor. These factors play a catalytic role in the wound healing cascade by activating neutrophils, and subsequently macrophages, endothelial cells and fibroblasts (Ellis et al., 2018). Platelets also contain vasoactive amines, such as histamine and 5-hydroxytryptamine (5-HT), which cause vasodilation and increased vascular permeability (Cañedo-Dorantes and Cañedo-Ayala, 2019). This phase lasts for minutes to hours and leads to an inflammatory phase.

### 2.2 Inflammatory phase

The purpose of the inflammatory response is to establish an immune barrier system against microbial invasion, removal of injury-causing factors and necrotic tissue. The inflammatory phase is characterized by infiltration of immune cells such as neutrophils, macrophages and lymphocytes (Wilkinson and Hardman, 2020). Neutrophils clean cellular debris and bacteria, providing a well infiltrated wound site for wound healing (Futosi et al., 2013). Secondly, highly abundant macrophages destroy and remove bacteria, foreign particles and damaged tissue. The phagocytosis products trigger the release of cytokines, growth factors, reactive oxygen species (ROS) and protein hydrolases, which in turn attract more immune cells to the site of injury (Zhao et al., 2016). During wound healing, the wound is populated by two macrophage subpopulations, pro-inflammatory (M1) and anti-inflammatory (M2) (Koh and DiPietro, 2011). The pro-inflammatory or anti-inflammatory phenotype of macrophages is tightly controlled by signaling pathways (Wilkinson and Hardman, 2020). Finally, the cells that enter the wound are lymphocytes, which are attracted to interleukin-1 (IL-1), complement components, and immunoglobulin G (IgG) breakdown products 72 h after injury (Wang et al., 2014). IL-1 plays an important role in the regulation of collagenase, which plays an important role in collagen remodeling, production of extracellular matrix components and their degradation. This phase begins immediately after injury, usually lasts four to 6 days, and is often accompanied by edema, erythema (reddening of the skin), inflammation, and pain.

### 2.3 Proliferative phase

The proliferative phase is characterized by the accumulation of large numbers of cells and connective tissue leading to epithelial closure, reconstruction of damaged areas, and tissue regeneration (Midwood et al., 2004). Epidermal growth factor (EGF) initiates re-epithelialization of the epidermis, stimulating keratin formation and cell protrusion, adhesion, contraction, and detachment (Ilina and Friedl, 2009). Angiogenesis is the formation of new capillaries in blood vessels, which grow mainly from small veins near the injury. Angiogenesis contributes to cell proliferation, migration, and collagen production (Betz et al., 2016). Stimulation by vascular endothelial growth factor (VEGF) involves three major steps, namely, endothelial cell movement, differentiation and maturation. First, in response to a vascular stimulus, endothelial cells produce certain proteases that degrade to the basement membrane of the vessel on the stimulated side. After about 24 h, the endothelial cells move across the basement membrane toward the stimulus and begin to divide and proliferate, forming solid bundles of cell strips. As a result of the maturation of the endothelial cells and the impact of blood flow, the middle portion of the neoplastic cell strip bundles opens up, and blood flow enters therefrom, forming newborn capillaries (Sorg et al., 2017). Together, the new capillaries and proliferating fibroblasts are known as granulation tissue, which is necessary for connective tissue repair. Fibroblasts and myofibroblasts migrate to the wound site and rebuild new tissue. Fibroblasts are responsible for synthesizing extracellular matrix (ECM) and collagen, which is an essential protein for maintaining the structural integrity of tissues (Wilkinson and Hardman, 2020). Myofibroblasts are specialized in migrating cells that help bring the edges of injured skin closer together. The proliferative phase usually lasts for days and weeks.

### 2.4 Remodeling phase

Remodeling is the final phase of wound healing and is responsible for the development of new epithelium and eventual scar tissue formation. The remodeling phase is characterized by the deposition of collagen, rearrangement of fibers and contraction of scar tissue (Tracy et al., 2016). The remodeling phase requires a precise balance between apoptosis of existing cells and the production of new cells. Extracellular matrix synthesis during the proliferation and remodeling phases is initiated concurrently with granulation tissue development and can last for several years (Velnar and Bailey, 2009). The gradual degradation of large amounts of extracellular matrix (ECM) and immature type III collagen during the remodeling phase and the gradual formation of mature type I collagen lead to an increase in the tensile strength of the newly formed tissue (Darby et al., 2014; de Oliveira Gonzalez et al., 2016; Wang et al., 2024). The newly formed fibers and collagen structures are relatively heterogeneous and may take years to reorganize correctly. In particular, the differentiation of fibroblasts into myofibroblasts contributes to the reduction of wound size. Once wound contraction is complete, apoptosis occurs in the population of immune cells, blood vessels and myofibroblasts (Hinz and Gabbiani, 2003; Di Pietro, 2013).

The remodeling phase of the healing process is very complex, and any aberrations at this stage can lead to excessive wound healing or chronic wounds (Gabbiani, 2003). It is often influenced by local and systemic factors, including infection, local maceration, age, nutritional status, and body size (whole body), all of which have a relationship with healing (Růžička et al., 2021). Bacterial infections often lead to the formation of exudates and biofilms that exhibit persistent abnormal inflammation and the development of a number of post-infectious complications, thus hindering the wound healing process. Hypoxia inhibits the wound healing process and increases the risk of infection by blocking fibroblast proliferation, collagen production, and capillogenesis. This phase lasts for months or years.

## 3 Properties and synthesis of CeO_2_ NPs

### 3.1 Properties of CeO_2_ NPs

**FIGURE 1:**
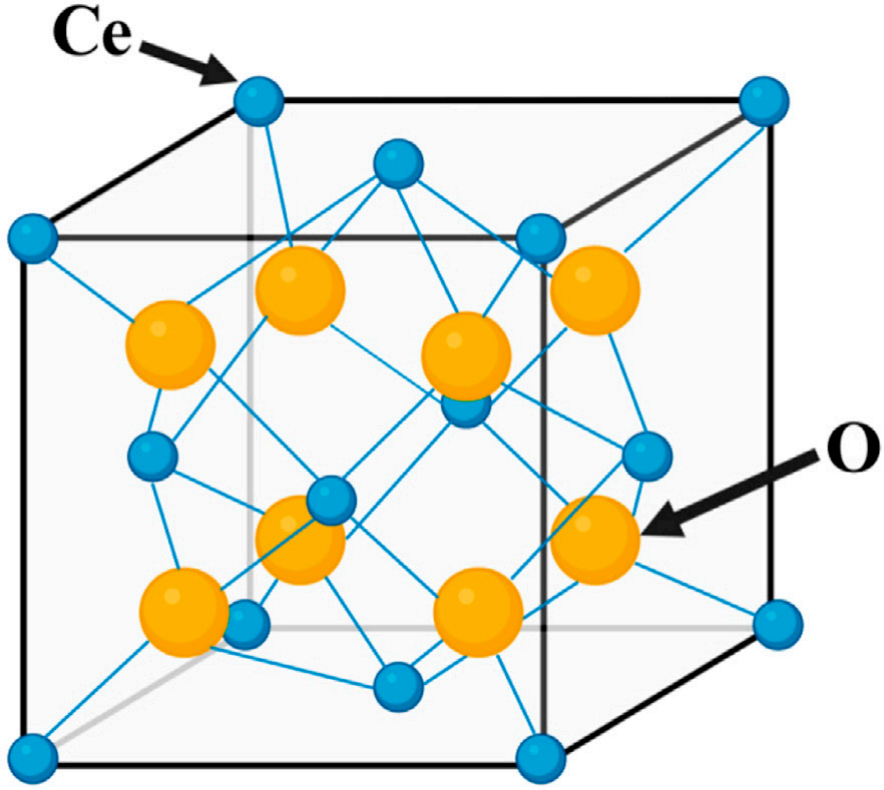
Crystal structure diagram of CeO_2_.

CeO_2_ generally occurs as the fluorite phase, with a face-centered cubic (fcc) crystal structure (Figure 1). Each cerium ion coordinates with eight oxygen ions. The electronic structure of cerium ([Xe]4f15d16s2) enables the reversible charge transfer between Ce^4+^ and Ce^3+^ states (Mehta et al., 2020). The properties of CeO_2_ are mainly influenced by its cubic fluorite lattice structure, which has many oxygen vacancies. These vacancies enable Ce oxides to possess both reduced CeO_2_ (Ce^4+^) and oxidized CeO_2_ (Ce^3+^) states (LI et al., 2018; Özkan et al., 2020). Ce^3+^ and Ce^4+^ can undergo interconversion during redox reactions, exhibiting activities that mimic superoxide dismutase (SOD) and catalase (CAT) (Celardo et al., 2011a; Kargozar et al., 2018). A high surface ratio of Ce^3+^/Ce^4+^ effectively catalyzes SOD mimic activity, whereas a low surface ratio of Ce^3+^/Ce^4+^ catalyzes CAT mimic activity. By controlling the Ce^4+^/Ce^3+^ ratio on the surface of CeO_2_ nanoparticles, it is possible to eliminate reactive oxygen species (ROS), which protects cells from oxidative stress damage and maintains a balance between macrophage anti-inflammatory and pro-inflammatory cytokines, thereby creating an anti-inflammatory microenvironment (Wang et al., 2015).

### 3.2 Synthesis of CeO_2_ NPs

**FIGURE 2:**
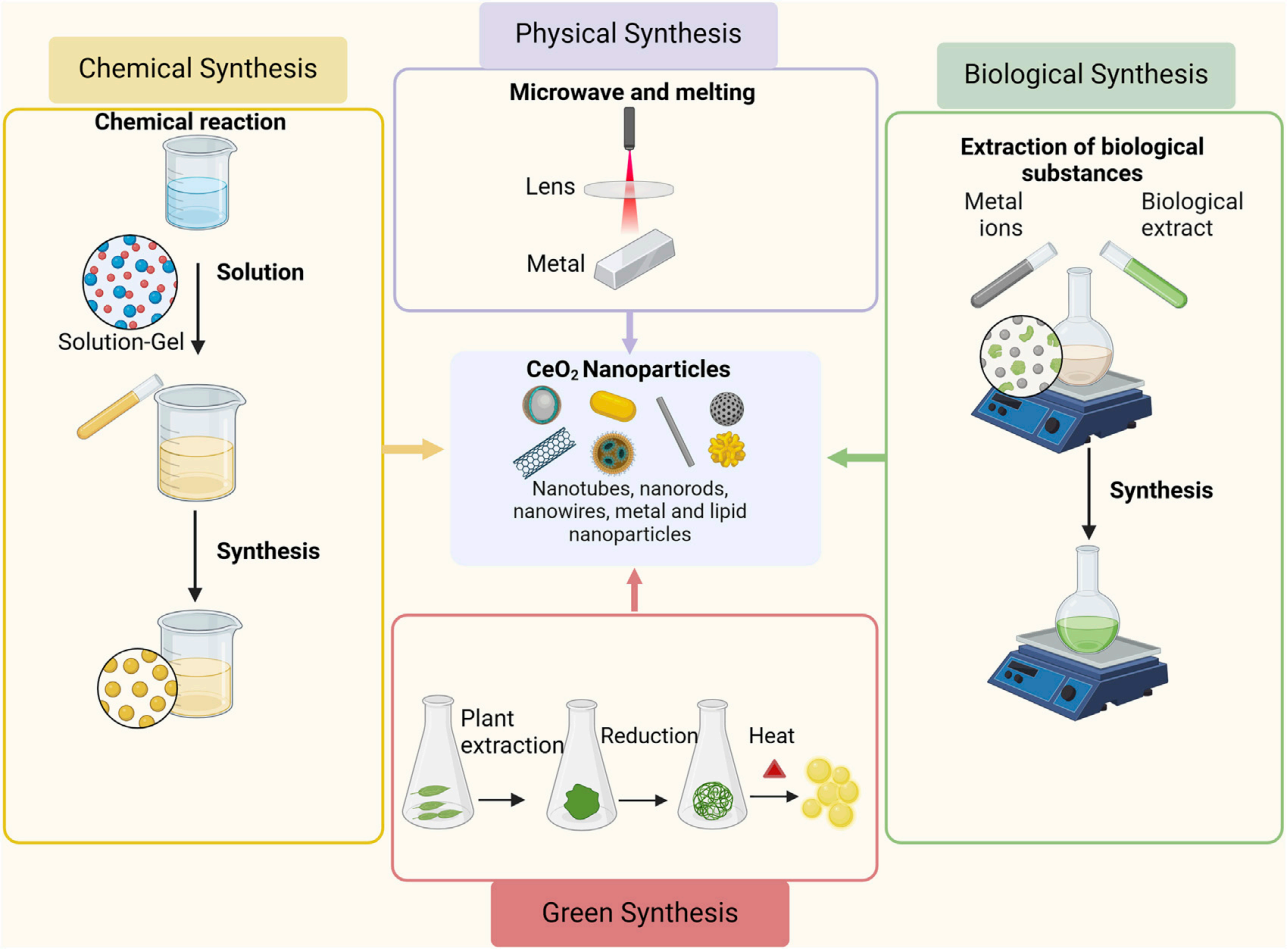
Synthesis methods of CeO_2_ NPs. Adapted from “Basic Methods of Nanoparticle Synthesis”, by BioRender.com (2020).

The synthesis of CeO_2_ can be classified according to various principles. For instance, depending on the conditions of powder synthesis, the methods can be divided into gas-phase, solid-phase, and liquid-phase methods. Furthermore, they can be classified as physical or chemical methods based on the underlying reaction mechanisms. Physical methods the milling methods, detonation, microwave, melting, and combustion, while chemical methods involve precipitation, sol-gel, hydrothermal, and electrochemical methods (Darroudi et al., 2014a; Charbgoo et al., 2017a; Nadeem et al., 2020). The method of synthesizing the nanoparticles is represented in Figure 2. Each method has its own advantages and limitations, which should be carefully considered when selecting a synthesis method (Nosrati et al., 2023). The milling methods is a fast, environmentally friendly, repeatable, and cost-effective executable technology, but there are also some problems, such as the possibility of environmental pollution, the tendency of nanoparticles to agglomerate, the formation of irregularly shaped nanoparticles, and the long time required for milling and cleaning (Naidi et al., 2021). Precipitation is the most commonly used method for the synthesis of CeO_2_ NPs, which produces nanoparticles with smaller size distributions and higher purity, but is also more complex and time-consuming (Sun et al., 2021). The sol-gel method is a versatile technique that can be used to synthesize CeO_2_ NPs of various sizes and shapes. It also allows precise control of the composition and purity of the nanoparticles. However, the process can be time-consuming and requires careful control of reaction conditions (Hosseini et al., 2020). The combustion method is a quick and easy method. However, this method requires careful control of experimental conditions to avoid unnecessary side reactions and obtain nanoparticles with the desired properties (Ghahramani et al., 2020).

CeO_2_ NPs synthesized using different methods exhibit distinct characteristics. The unique morphologies (e.g., nanocubes, nanorods, octahedra) and exposed crystal faces (e.g (111), (110), (100)) of nanoceria enable convenient synthesis and investigation of the structure-activity relationships in catalysts (Mai et al., 2005; Xu et al., 2021). Studies indicate that rod-shaped CeO_2_ NPs possess greater safety and efficacy as antioxidants (Ozkan et al., 2020). Additionally, research suggests that the crystal morphology and size of nanoceria may play pivotal roles in its cytotoxicity (de Oliveira et al., 2020). Certain sizes of CeO_2_ NPs demonstrate elevated reactivity and interact strongly with the nanostructure and metal substrates (Castano et al., 2015; Huang et al., 2018). Size additionally impacts the enhancement of electronic conductivity, pressure-induced phase transitions, size-induced lattice relaxation, and blue shift observed in the ultraviolet absorption spectra of CeO_2_ NPs (Singh et al., 2018). Size can either restrict or enhance the cellular uptake of CeO_2_ NPs while influencing biological parameters such as biologic half-life, diffusivity, immunogenicity, as well as *in vivo* and *in vitro* toxicity (Dahle and Arai, 2015; Eriksson et al., 2018a).

The choice of synthesis conditions has a significant impact on the final product. This means that nanomaterials produced using different methods will possess distinct physical and chemical properties. It is essential to identify the synthesis methods, particularly when considering their applications in medical fields, as the properties of nanoparticles directly influence their interactions with biological interfaces. Conventional physical and chemical methods used for CeO_2_ synthesis often involve the use of toxic solvents or reagents, as well as the addition of external stabilizers or capping agents, which can reduce the biosafety of CeO_2_. For instance, in chemical synthesis methods, toxic substances may adsorb onto the surface of nanoparticles, potentially causing adverse effects in the biological environment. Therefore, the adoption of novel synthesis methods for obtaining CeO_2_ is particularly crucial for its biomedical applications.

The green synthesis of CeO_2_ NPs has attracted attention due to their abundance, biocompatibility, and environmental friendliness (Charbgoo et al., 2017a). The main methods for green synthesis of CeO_2_ NPs include plant-mediated, fungus-mediated, polymer-mediated, and nutrient-mediated synthesis (Darroudi et al., 2014a). In plant-mediated methods, plant extracts function as stabilizers and capping agents, resulting in the production of relatively larger CeO_2_ NPs (Eriksson et al., 2018b; Darroudi et al., 2014b; Nosrati et al., 2023), which are currently unsuitable for biomedical applications (Charbgoo et al., 2017b). The plant-mediated methods demonstrates the advantages of high speed, High speed, eco-friendly, pollutant- and toxicity-free, more cost-effective (no cost for culture media and microorganism isolation), simple handling, stable and non-aggregated NPs, scalability, while also not required performing genetic manipulation like microorganisms (Zuhrotun et al., 2023). Fungus-mediated methods (fungal synthesis) address this problem by producing smaller CeO_2_ NPs (Kargar et al., 2015), which exhibit higher stability, water dispersibility, and fluorescence properties (Nyoka et al., 2020). Polymer-mediated CeO_2_ NPs offer advantages in terms of manageability, cost-effectiveness, and the utilization of less time-consuming and energy-consuming techniques. Biologically oriented synthesis of CeO_2_ using natural substrates as stabilizers provides a safer approach to preparing CeO_2_ NPs, thereby alleviating concerns regarding biocompatibility (Naidi et al., 2021). An example of this is the production of water-dispersible nanopowders using PEG in aqueous solutions (Sun et al., 2021). The synthesis of CeO_2_ NPs using nutrients is highly cost-effective (Charbgoo et al., 2017a; Seal et al., 2020). For example, when egg white is used as a substrate for synthesizing CeO_2_ NPs, the egg white proteins act as stabilizers, resulting in controlled isotropic growth of small CeO_2_ NPs (Johnson and Wilgus, 2014). The development of various methods for the green synthesis of CeO_2_ NPs enables them to enhance both the speed and quality of wound healing. In particular, CeO_2_ NPs synthesized through green methods have demonstrated both promising antibacterial and excellent antioxidative potential *in vitro* (Das et al., 2014). However, the exact mechanisms, toxicity, *in vivo* studies, and environmental concerns related to these nanoparticles remain unresolved despite their promising potential.

## 4 General properties of CeO_2_ nanoparticles for wound healing

CeO_2_ NPs have been shown to exhibit various biological properties, which contribute to their extensive utilization in the field of biomedical applications. CeO_2_ NPs have been shown to facilitate wound healing through the mitigation of inflammation, reduction of oxidative stress responses, mitigation of infection risks, and promotion of angiogenesis throughout the wound healing process (Walkey et al., 2015). These attributes render CeO_2_ NPs a promising candidate for application in wound healing.

### 4.1 Promoting angiogenesis

**FIGURE 3:**
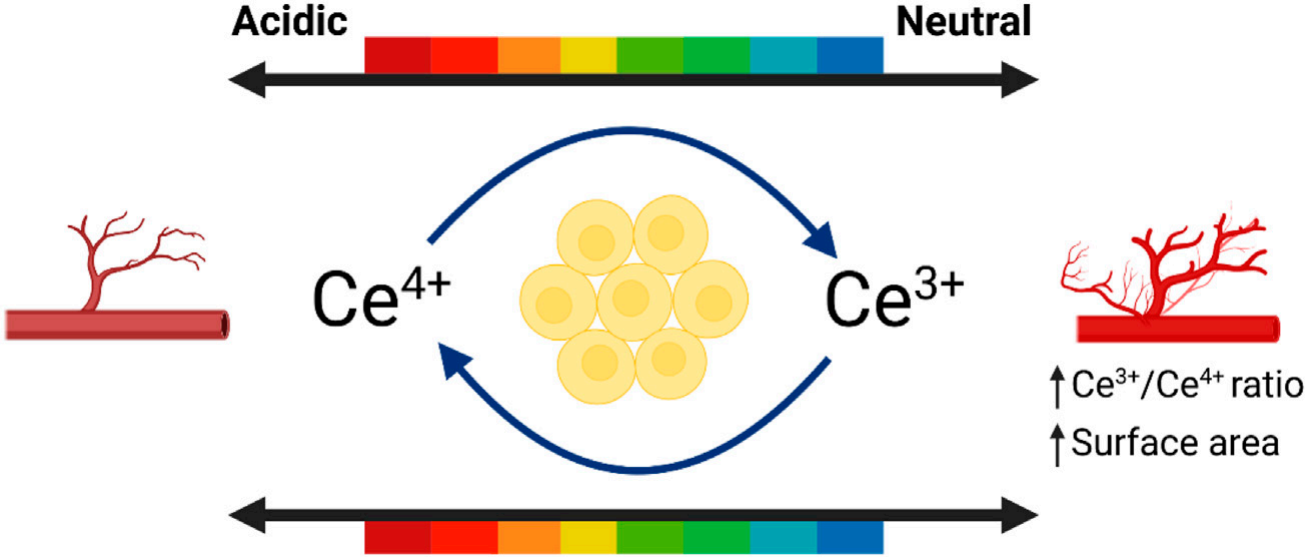
The influence of environment on promoting angiogenesis. Referenced and reproduced with permission (Xue et al., 2024).

CeO_2_ NPs can both promote and inhibit the new blood vessels (Das et al., 2012a; Chigurupati et al., 2013; Cheng et al., 2021a). Studies have shown that CeO_2_ NPs with diameters ranging from 3–5 nm and a higher surface Ce^3+^/Ce^4+^ ratio can stimulate the formation of vascular endothelial cell tubes *in vitro*. Smaller nanoparticles (≤15 nm) and higher surface Ce^3+^/Ce^4+^ ratios strongly induce angiogenesis (Figure 3). It has been observed that CeO_2_ NPs induce angiogenesis by modulating the intracellular oxygen environment. A correlation was found between the ability of nanoparticles to decrease intracellular oxygen levels and their potential to induce angiogenesis (Popova et al., 2020). Researchers have further investigated the impact of CeO_2_ NPs on the wound healing process due to their ability to modulate angiogenesis. Furthermore, CeO_2_ NPs promote tissue regeneration by alleviating oxidative stress. Importantly, CeO_2_ NPs may exhibit anti-angiogenic properties depending on the microenvironment, possibly due to their pH-dependent activity (Descamps and Emanueli, 2012).

**FIGURE 4:**
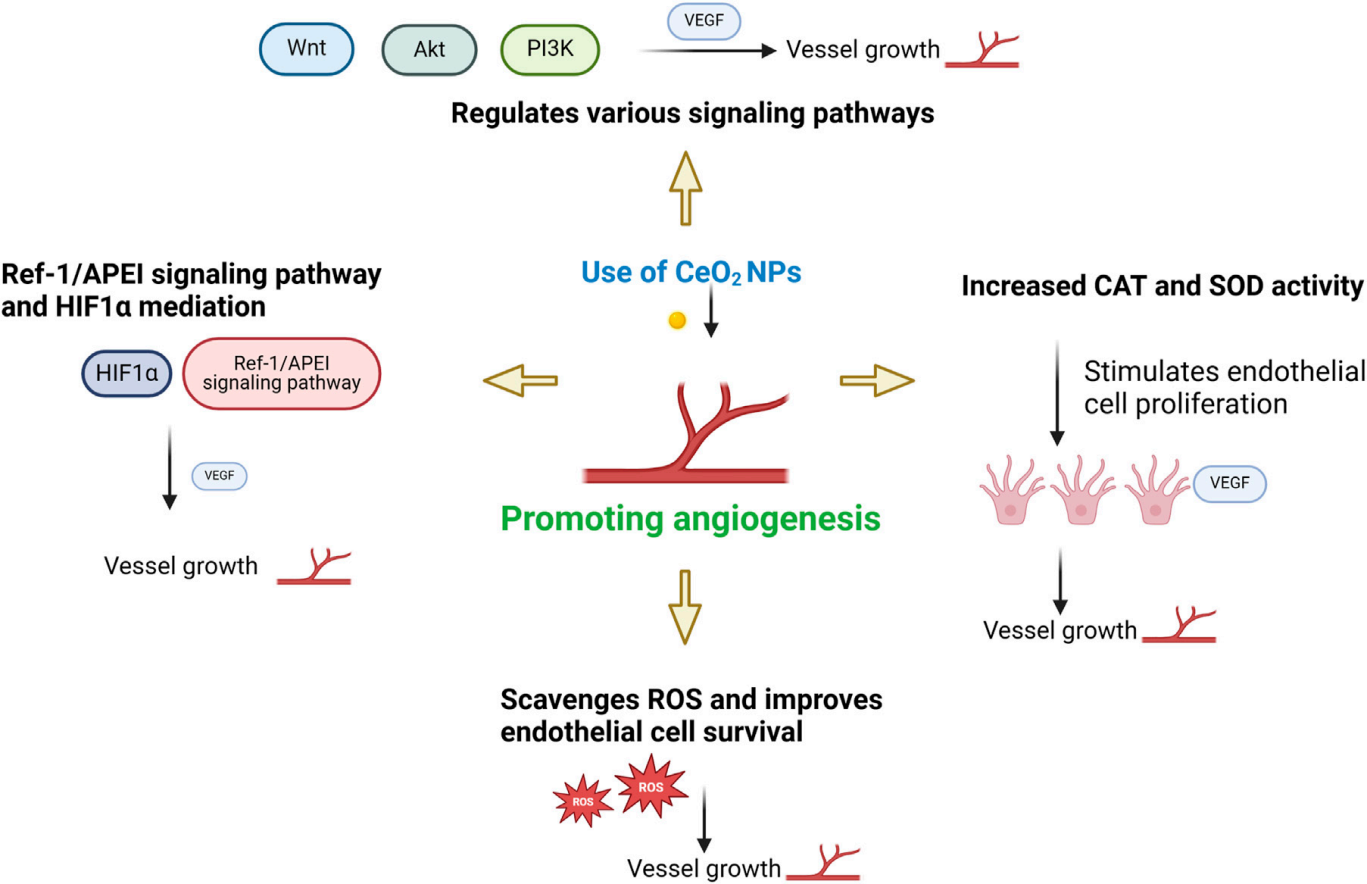
Mechanism of CeO_2_ NPs promoting angiogenesis.

CeO_2_ NPs can also induce vasoconstriction, activate thrombin, and facilitate platelet aggregation. CeO_2_ NPs regulate multiple signaling pathways, including PI3K/Akt, ERK/MAPK, and Wnt/β-catenin, to activate cell proliferation and growth. CeO_2_ NPs promote cell proliferation and growth through the regulation of these signaling pathways. Moreover, they enhance the activities of catalase (CAT) and superoxide dismutase (SOD), which safeguards cells from damage and facilitates wound healing. Additionally, they stimulate angiogenesis and endothelial cell proliferation, while enhancing nutrient supply to the wound site (Das et al., 2007). Research has demonstrated that CeO_2_ NPs facilitate the transportation of oxygen and nutrients to the injured site through vascular endothelial growth factor (VEGF) (Gopinath et al., 2015). The mediation of CeO_2_ NPs is facilitated by Ref-1/APEI signaling and the HIF-1α pathway. The decrease in oxygen levels temporarily triggers the nuclear translocation of hypoxia-inducible factor 1α (HIF-1α). Consequently, this stimulation leads to the expression of numerous proteins implicated in angiogenesis. Through its association with HIF-1α activation, the Ref-1/APEI signaling pathway provides direct support for the angiogenic effect of CeO_2_ NPs. The viability and angiogenesis of HUVECs were observed to be correlated with the upregulation of HIF-1α expression (Ludin et al., 2014; Abdal Dayem et al., 2017). In addition, CeO_2_ NPs counteract high levels of oxidative stress by eliminating ROS and promoting the survival of endothelial cells. Over time, this process fosters cellular generation and various endothelial cell functions, ultimately facilitating tissue repair, regeneration, and expediting the wound healing process (Damle et al., 2019; Ahmad et al., 2023). The mechanism by which cerium oxide nanoparticles promote angiogenesis is shown in Figure 4.

### 4.2 Redox activity

CeO_2_ NPs demonstrate excellent antioxidant properties, which aid in alleviating oxidative stress and inflammatory responses, thus promoting the wound healing process. Antioxidation safeguards cells from oxidative stress by allowing Ce^3+^ and Ce^4+^ oxidation states to fulfill antioxidative and pro-oxidative roles. The antioxidative effects of CeO_2_ NPs primarily stem from their redox capability, which is attributed to the abundance of Ce^3+^ and Ce^4+^ ions on their surface. These ions continuously interconvert, absorbing or releasing oxygen molecules as part of a redox cycle. Consequently, a redox bipolar layer forms on the surface of CeO_2_ NPs, offering cellular protection during oxidative stress and inflammatory responses (Cheng et al., 2021b). Additionally, CeO_2_ NPs can activate the Nrf2 signaling pathway to enhance the production and release of cellular antioxidants, thereby increasing cellular antioxidant capacity. CeO_2_ NPs also act on endothelial cells. In endothelial cells, CeO_2_ NPs did not affect cell viability, reduced oxidative stress, and inhibited mRNA-TF expression, VCAM-1 expression and cytokine release (Del Turco et al., 2019). All these remarkable features rely on the Ce^3+^/Ce^4+^ redox reaction (Naganuma, 2017).

**FIGURE 5:**
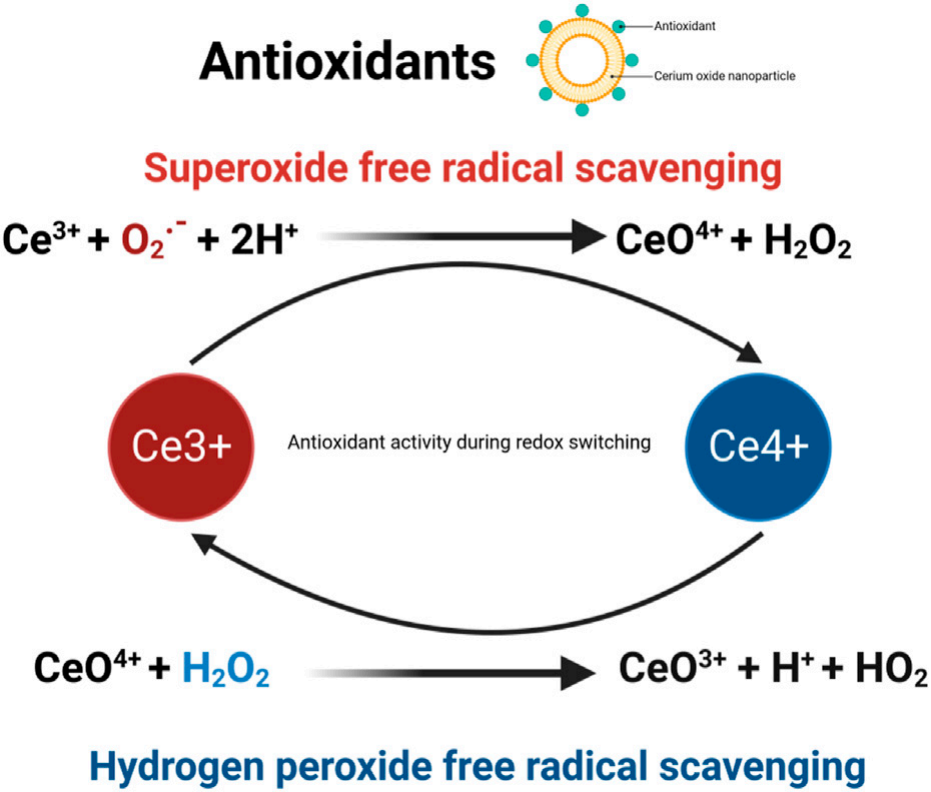
Antioxidation mechanism of cerium oxide nanoparticles.

Additionally, CeO_2_ NPs demonstrate outstanding enzymatic mimicry. They possess the ability to eliminate ROS (such as superoxide anion radicals) and reactive nitrogen species (such as nitric oxide radicals). Studies have demonstrated that the enzymatic activities of CeO_2_ NPs mimic antioxidants in a concentration-dependent manner, as evaluated by the DPPH radical scavenging test, superoxide dismutase (SOD) mimic activity test, and catalase mimic activity test. Catalase mimic activity measurements revealed that the activity increased until a concentration of 1,000 μg/mL of CeO_2_ NPs was reached (Ighodaro and Akinloye, 2018). Catalase (CAT) and superoxide dismutase (SOD) are enzymes with antioxidative properties, and one of the capabilities of CeO_2_ NPs is to eliminate ROS. Through the increase in catalase (CAT) mimic activity using Ce^4+^ ions and superoxide dismutase (SOD) mimic activity using Ce^3+^ ions (Burello and Worth, 2011; Kaarniranta et al., 2019; Lu et al., 2016), ROS can be eliminated. ROS, as signaling mediators, regulate cell growth, proliferation, differentiation, apoptosis, and autophagy (Nelson et al., 2016; Dutta et al., 2022). However, elevated levels of ROS can induce oxidative stress responses and contribute to pathogenesis (Datta et al., 2020). Additionally, CeO_2_ NPs can scavenge ROS at high concentrations and acidic pH, while reducing radicals at low concentrations and neutral pH (Khurana et al., 2018). Therefore, the antioxidant properties of CeO_2_ NPs depend on the concentration and pH value. Moreover, CeO_2_ NPs can undergo self-renewal and switch flexibly between antioxidative and pro-oxidative states (Liu et al., 2020). Pro-oxidative properties dominate at lower pH values (Clark et al., 2011). Pro-oxidative systems increase oxidative stress, which ultimately leads to cell apoptosis (Celardo et al., 2011b). Numerous experimental data show that CeO_2_ NPs protect against cell apoptosis induced by oxidative stress (Augustine et al., 2020; Purohit et al., 2020). In fact, the antioxidant system serves as the foundation for anti-apoptotic and pro-survival actions. The antioxidative properties of CeO_2_ NPs catalyze the decomposition of ROS, promoting rapid wound healing (Zholobak et al., 2016; Sadidi et al., 2020). The antioxidant mechanism of cerium oxide nanoparticles is shown in Figure 5.

### 4.3 Antibacterial properties

**FIGURE 6:**
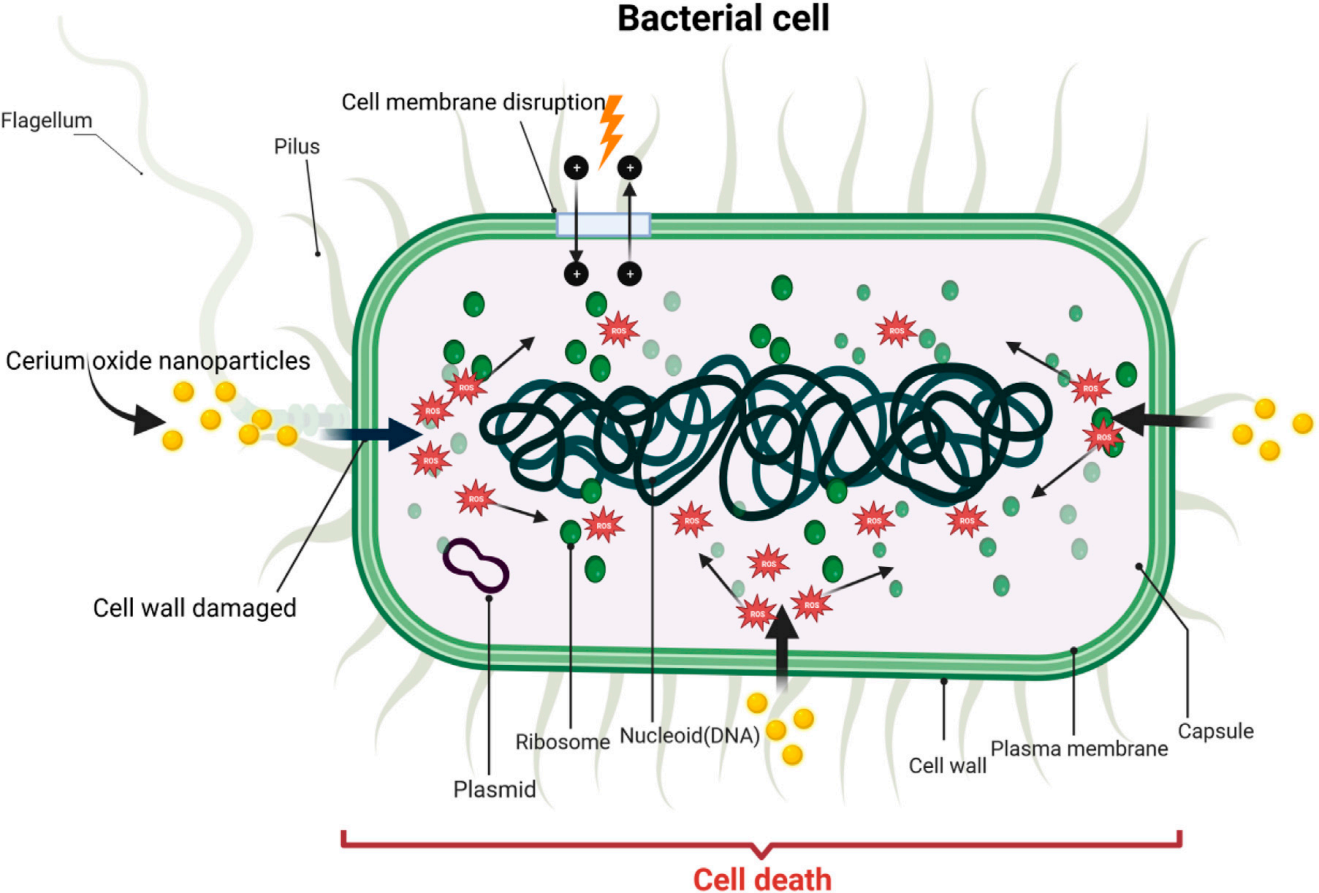
Antibacterial mechanism of cerium oxide nanoparticles.

CeO_2_ NPs exhibit antibacterial activity by inhibiting wound infections and bacterial growth, thereby reducing infection risks. The main antibacterial mechanism of CeO_2_ NPs involves direct contact with the bacterial membrane. Firstly, positively charged nanoparticles adsorb onto the negatively charged membrane of both Gram-negative and Gram-positive bacteria, causing them to adhere to the bacterial surface. This adsorption prevents the nanoparticles from penetrating the bacterial membrane, enabling them to persist on the bacterial surface for an extended period of time. Subsequently, these nanoparticles alter the viscosity of the membrane, disrupt the function of specific ion pumps, and significantly impact the exchange of substances between bacterial cells and the environment, thereby ultimately hindering bacterial growth (Babenko et al., 2012). Secondly, once CeO_2_ NPs are adsorbed onto the outer membrane of bacterial cells, they exert detrimental effects on proteins. One consequence of this interaction might be the release of cerium ions, which could disturb the bacterial electron flow and respiration (Li et al., 2012). Furthermore, these released ions might react with thiol groups (-SH) or be absorbed by transporters and/or pore proteins, potentially impeding nutrient transport (Das et al., 2012b). Moreover, the irregular shapes and rough edges of CeO_2_ NPs may induce physical damage to the bacterial membrane, particularly in Gram-positive bacteria. The main cause of nanomaterial toxicity in certain biological systems is the generation of ROS, which leads to oxidative stress (Park et al., 2020). ROS can induce the chemical breakdown of different organic components in microbes, leading to significant harm to bacteria. CeO_2_ NPs can also increase ROS levels in bacterial cells, potentially resulting in damage to DNA, RNA, proteins, lipids, and polysaccharides. Studies demonstrate the indirect interaction between CeO_2_ NPs and microbes enclosed in polysaccharides or forming biofilms, as CeO_2_ NPs are unable to directly penetrate the cell membrane (Chen et al., 2017). In this particular mechanism, the release of cerium ions from nanoparticle dissolution and the formation of ROS on the particle surface are of vital importance. ROS can target nucleic acids, proteins, polysaccharides, lipids, and other biomolecules, resulting in functional impairment and ultimately leading to the death and decomposition of bacteria. CeO_2_ NPs induce oxidative stress in the presence of bacteria (*E. coli*) by interacting with lipids or proteins within the microbial cells (Hirst et al., 2009). Hence, the antibacterial mechanism of CeO_2_ NPs is primarily mediated through the induction of oxidative stress by intracellular reactive oxygen species (ROS) (Sharma et al., 2020). The mechanism of antimicrobial activity of cerium oxide nanoparticles is shown in Figure 6.

### 4.4 Anti-inflammatory effects

The excessive and prolonged generation of ROS leads to oxidative stress, which contributes to the progression of inflammation and tissue damage. Therefore, because of their ability to scavenge ROS, CeO_2_ NPs are considered as anti-inflammatory agents. Studies have shown that CeO_2_ NPs can quench ROS in J774A cells and inhibit the production of iNOS. Hence, CeO_2_ NPs have potential therapeutic applications in the treatment of inflammatory diseases (Hirst et al., 2009). CeO_2_ NPs possess the ability to clear ROS, suppress inflammation, reduce cytokine levels, and protect cells both *in vivo* and *in vitro*. By inhibiting oxidative stress and activating the NF-κB signaling pathway, CeO_2_ NPs can reduce apoptosis and the release of inflammatory mediators, thereby lowering inflammation. Additionally, CeO_2_ NPs can inhibit the activity of epigenetic modifying enzymes, further reducing inflammatory responses. The anti-inflammatory effect of cerium oxide nanoparticles on endothelial cells plays a key role in wound healing and promotes rapid wound repair, Gojova A et al. incubated with CeO_2_ NPs at different concentrations (0.001–50 μg/mL) for 4 h, and subsequently measured the mRNA levels of three inflammatory markers, intercellular ad HAECs hesion molecule 1 (ICAM-1), interleukin (IL)-8, and monocyte chemotactic protein (MCP-1), using real-time polymerase chain reaction (PCR). (IL)-8 and monocyte chemotactic protein (MCP-1) mRNA levels. Even at the highest dose, CeO_2_ NPs rarely induced an inflammatory response in HAEC. This material is apparently harmless compared to Y (_2_)O (_3_) and ZnO nanoparticles that we have previously studied. These results suggest that the inflammatory response of HAEC after acute exposure to metal oxide nanoparticles is highly dependent on the particle composition. CeO_2_ NPs demonstrated excellent anti-inflammatory effects (Gojova et al., 2009). In another study, J. Ribera et al. evaluated the effects of albumin-coated 4 nm CeO_2_ NPs on primary endothelial cells isolated from the portal vein of cirrhotic rats and found that treatment with CeO_2_NPs reduced the pro-inflammatory state of endothelial cells, promoted an M2-like phenotype in macrophages in endothelial cell-conditioned media (antioxidant/regenerative), and reduced M1 polarization (pro-oxidant/defensive) (Ribera et al., 2019).

Research suggests that the distinctive valence state of CeO_2_ NPs serves as a scavenger for free radicals, enabling them to eliminate ROS or free radicals and suppress the generation of inflammatory mediators in macrophages (Saleh et al., 2020). They are also capable of downregulating the gene expression of pro-inflammatory cytokines (TSLP, Leukemia Inhibitory Factor (LIF), interleukin three and interleukin 7 (IL3 and IL7)), while increasing the expression of anti-inflammatory IL-6 and IL-13 (Kyosseva et al., 2013). Previous studies have demonstrated that CeO_2_ NPs do not induce an inflammatory response, even at high concentrations, such as 50 μg/mL (Wei et al., 2021). Some experimental data also indicate that CeO_2_ NPs have the potential to alleviate inflammation both *in vitro* and *in vivo*, thereby reducing the production of ROS in inflammatory conditions (Wang et al., 2018). Researchers have conducted *in vivo* studies, revealing that CeO_2_ NPs deposited in mouse tissues are non-pathogenic (Gagnon and Fromm, 2015; Bai et al., 2020; Ezhilarasu et al., 2020). Therefore, CeO_2_ NPs can alleviate inflammatory responses and decrease ROS production in inflammatory conditions.

**FIGURE 7:**
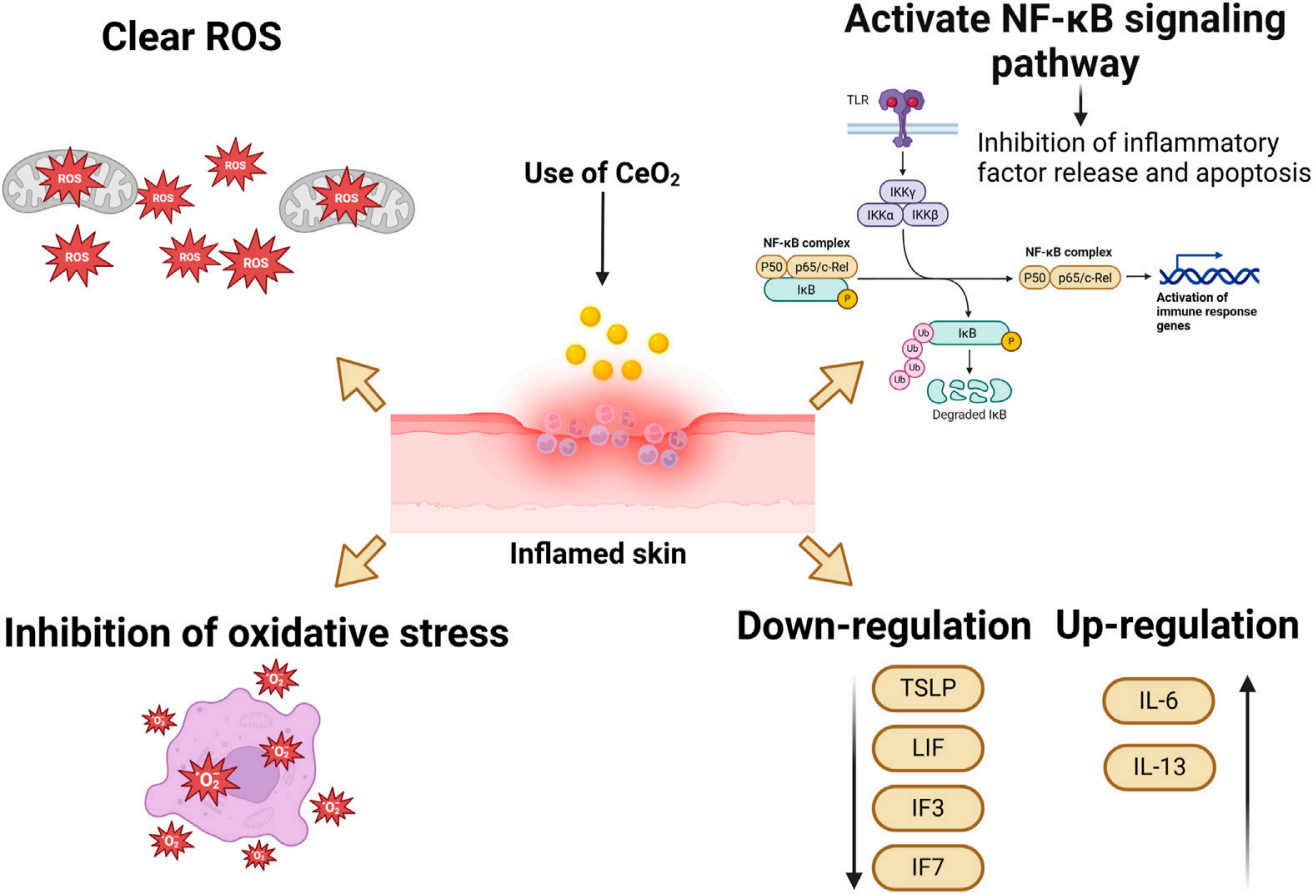
Anti-inflammatory mechanism of cerium oxide nanoparticles.

The anti-inflammatory mechanism of cerium oxide nanoparticles is demonstrated in Figure 7.

## 5 Applications of CeO_2_ in wound healing

Cerium oxide nanoparticles are often combined with other biomaterials for healing efficacy investigations. This integration is crucial for effectively releasing nanoparticles, extending drug release duration, and accelerating wound healing. Ultimately, the goal of this strategy is to expand the utilization of CeO_2_ in wound healing and improve treatment efficiency. In this article, we explore recent and relevant studies demonstrating the potential of CeO_2_ NPs conjugated polymeric scaffolds in wound healing applications. Specifically, the potential use of CeO_2_ NPs in repairing diabetic wounds has a significant impact on wound healing (Barbu et al., 2021; Chen et al., 2021; Stan et al., 2021).

### 5.1 CeO_2_ NPs with GelMA hydrogel

**FIGURE 8:**
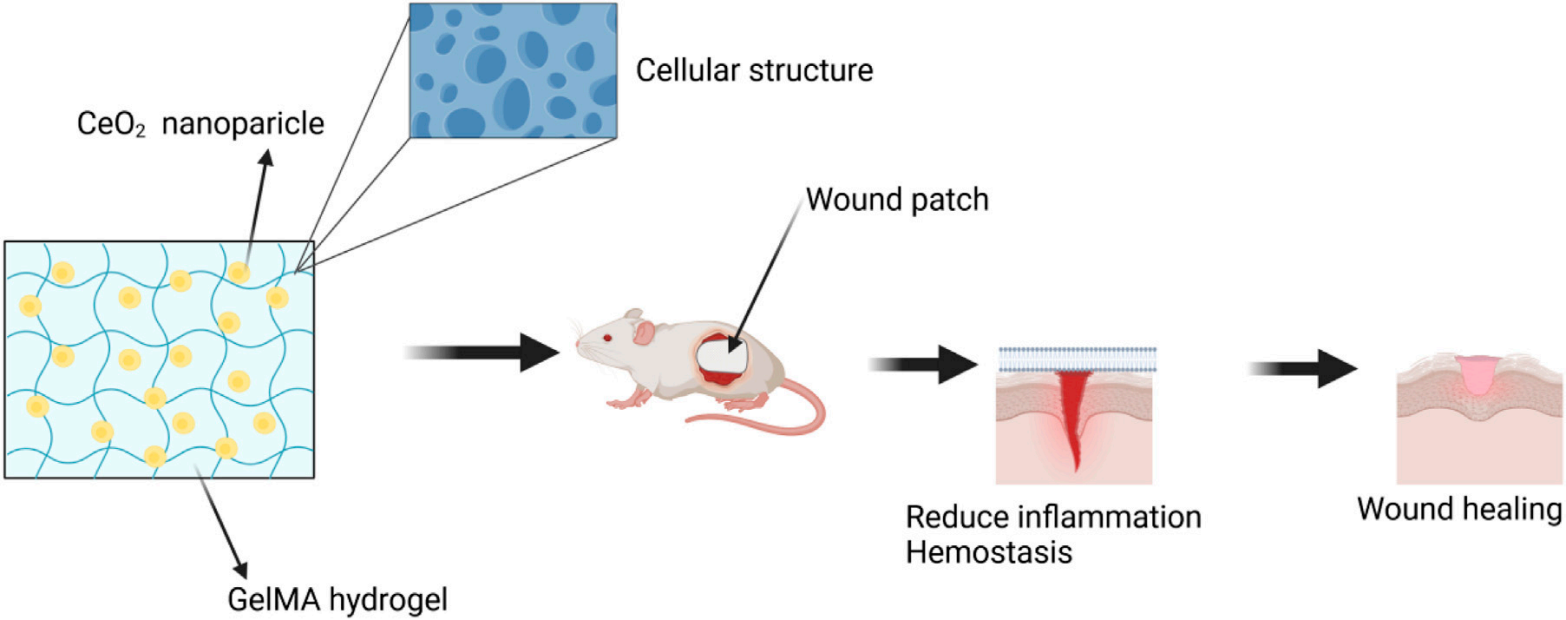
The wound was treated with a combination of CeO_2_ NPs and GelMA hydrogel. Referenced and reproduced with permission (Kyosseva et al., 2013).

A hydrogel patch made of biodegradable gelatin methacryloyl (GelMA), which contains CeO_2_ NPs, was developed to enhance the healing of diabetic wounds. A composite, injectable hydrogel consisting of cerium-containing bioactive glass (Ce-BG) and GelMA was prepared for the treatment of diabetic skin wounds. This composite hydrogel demonstrated multifunctional properties, including the inhibition of bacterial growth and enhancement of angiogenic activity, leading to an acceleration in the healing process of diabetic skin wounds (Augustine et al., 2021). Additionally, a wound healing patch composed of gelatin methacryloyl hydrogel loaded with CeO_2_ NPs was developed (Sener et al., 2020; Dewberry et al., 2022). The morphology, physical-mechanical properties, radical scavenging activity, *in vitro* cell proliferation, and *in vivo* diabetic wound healing activity of this patch were extensively analyzed. The highly porous and biodegradable patch exhibited excellent capability in absorbing exudate. In conclusion, the results indicate that the GelMA hydrogel loaded with CeO_2_ NPs has significant potential as a material for the development of therapeutic patches for the treatment of diabetic wounds, demonstrating good efficacy and promising prospects in wound healing. (Figure 8).

### 5.2 CeO_2_ NPs with microRNA-146a

**FIGURE 9:**
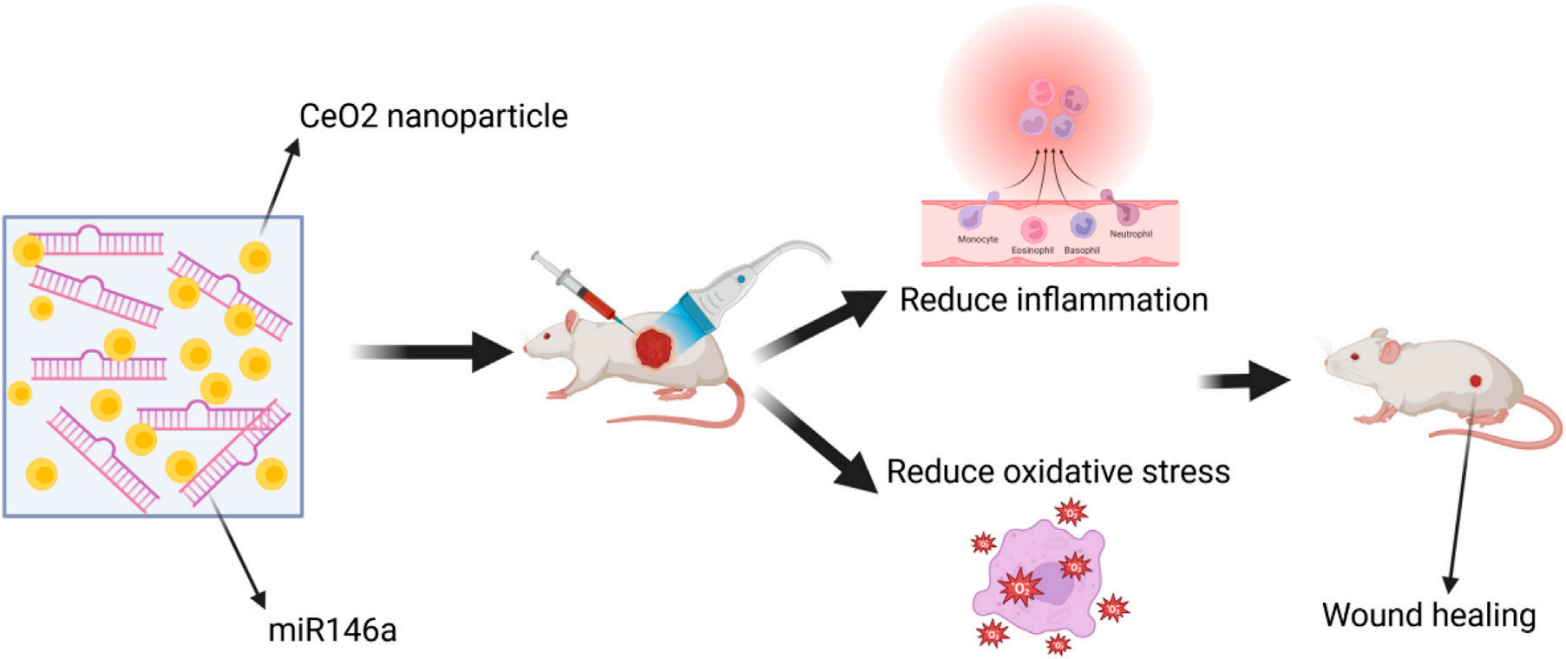
The wound was treated with a combination of CeO_2_ NPs and microRNA-146a.

The CNP-miR146a conjugate, consisting of CeO_2_ NPs and microRNA (miR)-146a, was found to enhance diabetic wound healing. CeO_2_ NPs, a divalent metal oxide, functions as an antioxidant by scavenging free radicals, and miR146a inhibits the pro-inflammatory NFκB pathway. Consequently, CNP-miR146a exerts a synergistic effect in regulating both oxidative stress and inflammation. Intradermal injection of CNP-miR146a was found to promote collagen production, enhance angiogenesis, reduce inflammation and oxidative stress, ultimately resulting in accelerated closure of diabetic wounds (Stager et al., 2022). Additionally, a biomaterial system based on an amphoteric ionic cryogel (gel formed at sub-zero temperatures) incorporating CNP-miR146a was developed. This system is suitable for local injection, self-healing, and provides sustained release of therapeutic molecules (Zgheib et al., 2019). The researchers utilized CeO_2_ NPs tagged with microRNA-146a (CNP-miR146a) to address oxidative stress and inflammation. In an *in vivo* diabetic mouse wound healing model, both the amphoteric ionic hydrogel alone and the hydrogel loaded with CNP-miR146a conjugate demonstrated significant improvements in diabetic wound healing rates (Niemiec et al., 2020). A dose of 100 ng of CNP-miR146a was found to improve diabetic wound healing without compromising the biomechanical properties of the healed skin (Rivera-Briso and Serrano-ArocaÁ, 2018). Moreover, research has shown that nano-silk can enhance the strength of diabetic skin, delivering CNP-miR146a to improve wound healing. The nano-fiber solution not only enhances the biomechanical properties of diabetic skin but also successfully delivers CNP-miR146a, thereby improving diabetic wound healing through the inhibition of pro-inflammatory gene signaling and the promotion of fibrogenic processes (Augustine et al., 2020). In conclusion, this nanotechnology-based therapy shows promise, and future research should focus on translating it into clinical applications (Figure 9).

### 5.3 CeO_2_ NPs with PHBV (Poly (3-hydroxybutyrate-co-3-hydroxyvalerate))

**FIGURE 10:**
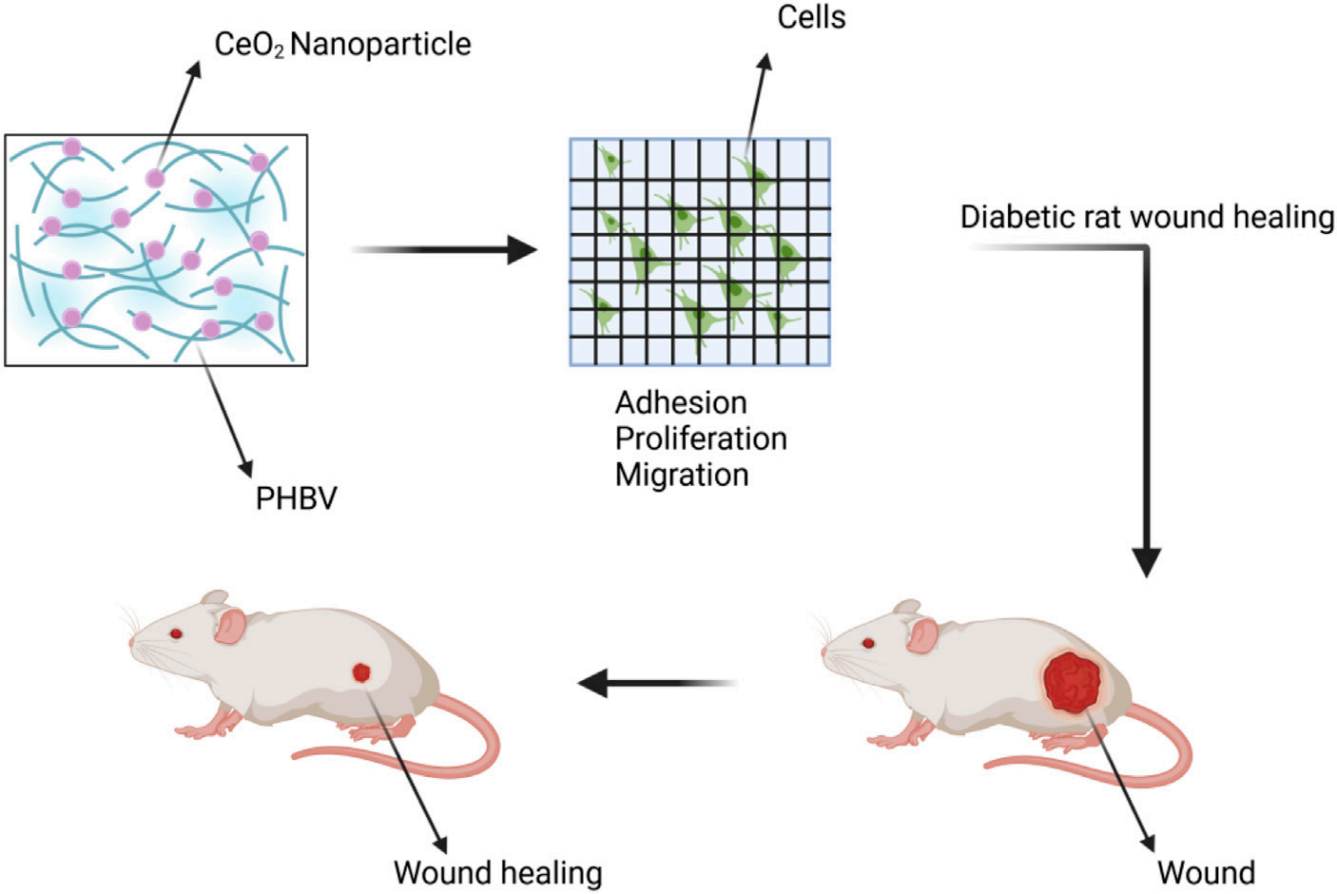
The wound was treated with a combination of CeO_2_ NPs and PHBV (Poly (3-hydroxybutyrate-co-3-hydroxyvalerate)).

PHBV(Poly (3-hydroxybutyrate-co-3-hydroxyvalerate)) is a biodegradable, non-toxic, and biocompatible polyhydroxyalkanoate polymer (Luo et al., 2023). Cell membranes made of PHBV and encapsulating CeO_2_ NPs have shown potential in the treatment of complications in diabetic wound healing. In in vivo experiments on diabetic rats, this membrane demonstrated the ability to repair wounds. We developed a novel membrane made of electrospun poly (3-hydroxybutyrate-co-3-hydroxyvalerate) (PHBV) containing CeO_2_ for applications in diabetic wound healing. We evaluated the membrane’s potential for cell proliferation, angiogenesis, and wound healing through *in vitro* cell adhesion studies, chick embryo angiogenesis assays, and *in vivo* experiments on diabetic wound healing. The results showed that the PHBV membrane containing CeO_2_ promoted cell proliferation and adhesion when used as a wound dressing, leading to improved healing of diabetic wounds (Aalapati et al., 2014). We prepared nanofibers using the electrospinning method, which were based on PHBV and CeO_2_ NPs. The electrospinning process resulted in the formation of highly porous nanofiber matrices, enabling the free diffusion of oxygen and nutrients. This facilitates rapid and successful wound healing (Sadidi et al., 2020). CeO_2_ NP containing PHBV promotes wound healing in Figure 10.

## 6 Recent advancements

In order to have a deeper understanding of CeO_2_ NP, researchers have actively studied and summarized new research progress on CeO_2_ NPs. Firstly, Pandey S et al. found that different temperature ranges had a strong effect on the redox activity of CeO_2_ NPs, and observed that the superoxide dismutase mimetic activity decreased with decreasing temperature. CeO_2_ NP were also found to inhibit α-amylase activity by up to 60% at a concentration of 1 mM, suggesting their potential application in anti-diabetic wound healing therapy. In conclusion, CeO_2_ NP have found application in mitigating oxidative stress-related diseases exhibited by their high antioxidant, antimicrobial and antidiabetic behavior (Pandey et al., 2024). Secondly, Bai Y et al. state that due to their excellent catalytic activities, CeO_2_ NP have promise as biological nanoenzymes. A redox reaction occurs between Ce^3+^ ions and Ce^4+^ ions during which they undergo conversion by acquiring or losing electrons as well as forming oxygen vacancies (or defects) in the lattice structure, which can act as antioxidant enzymes and simulate various enzyme activities. A number of CeO_2_ NPs have been engineered with multienzyme activities, including catalase, superoxide oxidase, peroxidase, and oxidase mimetic properties. CeO_2_ NPs have nitric oxide radical clearing and radical scavenging properties and have been widely used in a number of fields of biology, including biomedicine, disease diagnosis, and treatment (Bai et al., 2024). In addition, new advances have also been made in the molecular pathways involved in wound healing with CeO_2_ NP. He S et al. found NLRP3 pathway-targeting ability to mediate mitochondrial function recovery. DEGs significantly enriched in immune, metabolic and complement cascade related pathways, particularly in NLRP3 pathway. TNFR2 non-canonical NF-kB pathway and TNFs binding their physiological receptors pathways were also enriched. Specifically, though treated with CeO_2_–Y@ZIF-8@Gel, the Ox-mtDNA was either repaired or less escaped within mitochondria via mPTP-dependent channels to inhibit cytosolic NLRP3 inflammasome (a key sensor and effector of tissue damage) initiation and IL-1β secretion leading Macrophages polarization. Meanwhile, cGAS-STING pathway activation in Macrophages was reduced as less mtDNA was uptaken and Macrophages ultimately exhibited polarization to the anti-inflammatory phenotype (He et al., 2024). Conventional nanozymes have limitations in preventing the continuous production of ROS, including the most oxidizing reactive hydroxyl radical (·OH), although they can remove pre-existing ROS. Zhu Z et al. found that a novel antioxidant nanoplatform addresses this challenge by incorporating JSH-23 into the mesoporous of cupric-doped cerium oxide nanozymes. Additionally, for rapid wound adaptability and durable tissue adhesion, a nanozyme hydrogel spray consisting of oxidized sodium alginate and methacrylate gelatin is constructed, named OG@CCJs. This platform resurrects Nrf2 transcriptional activity of macrophages *in vitro*, curbing the production of ROS at its source, particularly ·OH, while enabling the nanozymes to scavenge previously generated ROS. OG@CCJs significantly alleviate oxidative stress in diabetic wounds *in vivo*, promoting wound healing. Overall, the proposed nanozyme-hydrogel spray with enhanced ·OH-scavenging activity uses a “two-track” antioxidant strategy to rebuild the antioxidant defense barrier of macrophages (Zhu et al., 2024). Carvajal S et al. showed that CeO_2_ NPs reverted the H_2_O_2_-mediated increase in the phosphorylation of peptides related to cellular proliferation, stress response, and gene transcription regulation, and interfered with H_2_O_2_ effects on mTOR, MAPK/ERK, CK2A1, and PKACA signaling pathways (Carvajal et al., 2019).

In conclusion, these latest researches will push the research of CeO_2_ NPs to be more perfect, and together they will promote the development of biomedicine, which will be more favorable to the development of nanoparticles in the future, and push more researchers to carry out researches to improve the nanoparticles.

## 7 Challenges and prospects

### 7.1 Toxicity and safety of CeO_2_ NPs

Although the potential of CeO_2_ NP in various biomedical applications has been demonstrated, there are still shortcomings in the existing studies (Rzigalinski et al., 2017; Maccarone et al., 2020). In terms of toxicity considerations, the toxicity of CeO_2_ NPs is affected by a variety of factors, including particle size, synthesis method, cell type, dose/concentration, exposure time and exposure route (Li et al., 2023). First, in terms of dose and concentration, CeO_2_ NPs are generally not toxic *in vivo* at therapeutic doses. However, excessive release of cerium ions and at high doses (>1 10th of a milligram of CeO2 per kilogram of animal) show toxicity. The rapid release of CeO_2_ NPs has the following potential toxicity issues: first, the rapidly released cerium oxide nanoparticles may have direct toxic effects on cells and tissues around the wound. These toxic effects may include oxidative stress, apoptosis, DNA damage, etc., leading to damage of cell structure and function. These damages may delay the wound healing process and even trigger complications such as inflammation and infection. Second, cerium oxide nanoparticles may also interact with biomolecules in the wound and interfere with normal biological processes. For example, they may bind to biomolecules such as proteins and DNA, affecting their function and expression. These interferences may negatively affect the wound healing process, such as affecting cell proliferation, migration and differentiation. In addition, rapidly released cerium oxide nanoparticles may trigger an immune response, leading to inflammation and activation of immune cells. Although a moderate immune response can help wound healing, an excessive immune response may lead to tissue damage and increased inflammation, thereby affecting wound healing. Therefore, the potential risks of cerium oxide nanoparticles in wound healing should be carefully evaluated in practical applications and appropriate protective measures should be taken to ensure patient safety. So determining the ideal concentration of CeO_2_ NPs that stimulates cell growth in natural tissues and accelerates the healing process is challenging and requires precise control of the surface chemistry, particle size, physicochemical properties, route of administration, and synthesis method of CeO_2_ NPs. The identification of the optimal concentration is similar to its consideration of the factors influencing the toxicity of CeO_2_ NPs. In terms of morphology, different morphologies have different toxicities; Ji et al. observed that CeO_2_ NPs (ranging in length from a few hundred nanometers to a few micrometers) induced a gradual increase in IL-1β production by producing lysosomal damage, whereas CeO_2_ nanospheres and shorter nanorods did not show significant toxicity (Ji et al., 2012). The time and route of exposure play an important role in the toxicity of CeO_2_ NPs, as shown by Carlander et al., who administered CeO_2_ NPs of different sizes, coatings, and dosages to rats via different routes of exposure, which showed that the biokinetics of CeO_2_ NPs are not only dependent on the properties of the NPs (size and coatings), but also on the conditions of the exposure (routes and dosages) (Carlander et al., 2018). Mackevica et al. found that extrapolations of results obtained under high exposure conditions may not be applicable to real-world environmental conditions, as higher NP concentrations may lead to increased NP-to-cell ratios, which can cause testing errors (Mackevica et al., 2023). Surface modifiers can also affect NPs, Fisichella et al. found that uncoated CeO_2_ NPs downregulated key genes involved in metabolic activity, whereas ammonium citrate-coated CeO_2_ NPs did not show any detrimental effects at the same concentration (Fisichella et al., 2014). From a cost consideration, CeO_2_ NPs, as a rare earth oxide metal, are expensive and costly to use compared to other wound healing materials and do not offer a price advantage, which economically hinders the possibility of nanomedicine becoming a reality. From a standardization point of view, CeO_2_ NPs research lacks uniform standards and there may be differences between studies that make it difficult to make comparisons. Cerium dioxide nanoparticles have similar safety concerns as other insoluble nanomaterials. For example, the incorporation of CeO_2_ into polymer matrices such as 3D scaffolds may improve biocompatibility, but may also reduce therapeutic efficacy (Singh et al., 2021; He et al., 2023). Therefore, harmonization of standards will help to improve the potential and safety of cerium oxide nanoparticles. A major drawback from intellectual and technical considerations is that the safety of nanomaterials is still a widely debated topic. Currently, cerium oxide nanoparticles have shown excellent wound healing ability in animal experiments. For example, animal experiments conducted by Zhao R et al. on a rat whole skin wound model showed that alginate hydrogel-based wound dressings were effective in accelerating the wound healing process. This study demonstrated the safety and reliability of CeO_2_ NPs (Zhao et al., 2023). However, despite the medical advantages of nanomaterials and the impressive research results, few nanomaterials can be used in clinical applications. Clinical trials related to cerium oxide nanoparticles are still blank. There is also a need to understand the precise evolution and biodistribution (ADME characterization) of NPs in the human body if they are to be used safely and effectively. In this context, the development of reproducible and reliable analytical methods for the dynamic characterization of the evolution of nanomaterials in biological environments is considered to be an important way to perform nanosafety studies. Therefore, for the future development of cerium oxide nanoparticles, in addition to innovations in animal experiments, more breakthroughs in human trials are needed in the future to better demonstrate the wound healing ability of cerium oxide nanoparticles.

### 7.2 Potential effects of cerium oxide nanoparticle composites

Difficulties arise in the use of single nanoparticles for wound healing, so investigating composite biomaterials for wound healing and addressing the challenges encountered with individual nanomaterials in the healing process could elucidate their unique advantages in promoting wound repair (Veith et al., 2019; Zeng et al., 2022). Efforts have been made to improve the effectiveness of CeO_2_ NPs at various stages of the wound healing process. Cheng et al. investigated and designed cerium-nitrogen-phosphorus-striped peelable graphene nanocomposites (abbreviated as ACGNCs) to enhance wound healing by promoting the inflammatory and proliferative phases. The ACGNCs exerted the following roles to promote wound healing: (1) in the inflammatory phase, the pH value was still acidic (∼6. 0), ACG NCs received white light irradiation and produced large amounts of ROS to scavenge bacteria due to the effective separation of electron-hole space; (2) as the wound healing process proceeded to the proliferative phase, the pH was naturally elevated at a neutral level, and the folded single-stranded DNA variant of i-motif DNA was transformed into an unfolded form, which facilitated the opening of the pore of the hollow cerium dioxide NPs to release parameters that It can be decomposed into urea with the help of arginase overexpression site in the wound; (3) the isolated cerium dioxide NPs can freely enter into the fibroblasts, thus destroying the intracellular ROS and promoting cell proliferation, and the stealth peptide prevents the graphene from being absorbed by the macrophage, prolongs the retention time of graphene as a scaffold in the wound site, and thus promotes the migration of fibroblasts. Finally, during the remodeling phase, MMP, which is highly expressed in the extracellular matrix, cleaves the GPLGLAG peptide and promotes the internalization of graphene by macrophages through endocytosis, which leads to the biodegradation of ACG nc at the wound site (Cheng et al., 2019). In the study of Ma et al. hollow CeO_2_ NPs with porous shells and rough surfaces were synthesized and L-arginine was added to them to promote various stages of wound healing. In the hemostatic stage, the modified CeO_2_ NPs acted as nanobridges within the tissue to achieve rapid hemostasis. In the inflammatory stage, these nanoparticles generate ROS under simulated sunlight irradiation to eliminate bacteria and prevent infection. In the proliferative phase, modified CeO_2_ NPs can scavenge excess ROS generated at the wound site due to SOD and catalase activities. In addition, the released L-arginine can be converted to nitric oxide in macrophages, thus promoting cell proliferation (Ma et al., 2019).

Secondly, it was also shown that combining CeO_2_ with polymers such as polycaprolactone (PCL), polyvinyl alcohol and chitosan does not affect the catalytic ability of enzymes. These composites have potential applications in wound healing, as confirmed by intracellular experiments. Based on the enzyme-catalyzing ability of CeO_2_, dressings with antimicrobial, anti-inflammatory and antioxidant properties have been developed. For example, when tested by Plocon C et al. using human osteoblasts, the composite scaffolds showed very good biocompatibility, with samples consisting of polymers and cerium-doped calcium phosphate responding better (Plocon et al., 2023). Sanmugam A et al. found that the fabricated scaffolds completely facilitated the complete closure of the wounds after 14 days, which suggests that the developed scaffolds have high swelling, good degradability and permeability, which facilitated the absorption of wound exudates, thus consolidating the healing effect (Sanmugam et al., 2024). Kamalipooya S et al. found that the nanocomposite with 0.1% CeO_2_-CSNPs exhibited high antibacterial performance against *S. aureus* (<58.59 μg/mL). The results of this research suggest that PCL/CA nanofiber mats functionalized with CeO_2_-CSNPs have the potential to be highly effective in treating diabetes-related wounds. The use of CeO_2_ NPs in diabetic wound repair substances has shown promising results (Kamalipooya et al., 2024). The popularity of combining nanoparticles with hydrogels is due to the highwater retention capacity of hydrogels, which provides a favorable microenvironment for cell proliferation, enabling the loading of bioactives and providing a soft texture and excellent wound healing properties. Hydrogels (including chitosan hydrogels, alginate hydrogels (Raja and Fathima, 2018) and silk-based hydrogels (Augustine et al., 2021)) have favorable physical properties and chemical structures. Numerous studies have shown significant progress in the utilization of hydrogels for sutureless wound closure, antimicrobial, hemostasis and angiogenesis. Hydrogels based on natural polymers have great potential for cell proliferation due to the presence of biomolecules and peptides, in addition to their general advantages. ZC-QPP hydrogels provide an avenue for the development of a multifunctional synergistic therapeutic platform that combines enzyme nanomaterials with hydrogels to synergize antimicrobial and antioxidant properties and promote wound healing (Zhang et al., 2023). CeO_2_ NPs contain Ce^3+^ ions and produce oxygen vacancies. The Ce^3+^ ions on the surface are the site of catalytic reactions, while the generated oxygen vacancies contribute to the conversion and migration of active substances in the system. All these factors will be favorable for biosensors (Soni et al., 2018; Domínguez-Aragón et al., 2021). The redox activity of cerium oxide can be used as a sensing platform to generate color in the presence of oxidative enzymes acting on the corresponding substrates to produce H_2_O_2_. CeO_2_ particles can be used both as a high specific surface area support for immobilizing highly loaded enzymes and as a reagent for generating color (Karimi et al., 2016).

In summary, CeO_2_ NPs, both naked and functionalized, show excellent ability to accelerate acute and chronic wound healing. CeO_2_ NPs can be used in wound dressings, drug delivery systems and biomedical nanotechnology to promote wound healing. They have antioxidant and antimicrobial properties that help reduce the risk of infection and promote tissue regeneration and repair. In addition, CeO_2_ NPs can be used as drug carriers to control the release of drugs to promote wound healing. They can also be used to fabricate biosensors to monitor biomarkers and inflammatory responses during wound healing for real-time diagnosis and therapeutic feedback (Sadidi et al., 2020; Xue et al., 2024). In addition to their wide range of applications in wound healing, CeO_2_ NPs are mainly used in the treatment of skin, cardiac, neurological and ophthalmic tissues. Other soft tissues may also be evaluated in future experimental studies. Thin films, hydrogels and nanofiber scaffolds containing CeO_2_ NPs have been shown to be highly suitable alternatives with satisfactory results in the repair and regeneration of soft tissue injuries and defects. Mesenchymal stem cell-derived extracellular vesicles (EVs) carried with CeO_2_ NPs shows promising in the field of regenerative medicine as they have a powerful role in promoting tissue repair and regeneration (Sadidi et al., 2020). However, the fragile lipid membrane limits their function in oxidative stress microenvironments. Cerium nanocerium is an antioxidant nanoenzymes; herein, we reveal that cerium nanocerium-loaded EVs extracted from MSCs promote skin wound healing in aged mice. Gao L et al. further demonstrated that the DG-CeO_2_ EVsHyp are biocompatible and have antioxidant and pro-angiogenic effects during skin wound healing in both young and aged mice (Gao et al., 2023). Yildizbakan L et al. found that synthetic chitosan-cerium oxide porous scaffolds are an effective solution to complications associated with bone injury by promoting tissue regeneration and reducing the risk of infection. All scaffold variants inhibited the bacterial growth of *Staphylococcus aureus* and *Escherichia coli* strains (Yildizbakan et al., 2023).

### 7.3 Potential of CeO_2_ NPs for interdisciplinary applications

We are looking forward to the interdisciplinary field of CeO_2_ NPs, which is widely used as a nanomaterial in wound dressings or other biomedical applications, a novel approach that has revolutionized the therapeutic regimen and management of wound healing. The unrivaled advantages of CeO_2_ NPs in wound care compared with other materials mainly include (1) high reactivity and large specific surface area with higher catalytic and gas production/loading efficiencies, and (2) control of the conductivity and penetration depth of the nanomaterials by changing the nanomaterials’ size and shape. However, the use of nanomaterials for wound healing is far from commercialization and clinical application. Major obstacles include: (1) nanomaterials have toxic effects, mainly in the form of oxidative stress and inflammation in cells, and in many cases, it is difficult to gather sufficient information about the expected behavior and toxicity of nanosystems in the human body; (2) nanomaterials have a single function in wound healing and prevention of infections, and insufficient ability in hemostasis, keeping wounds moist, isolating wounds, and promoting wound healing; (3) Nanomaterials are mainly injected into wounds *in situ*, producing liquids that are not only easy to spill out of wounds, but also pose challenges to clinical care.

In order to accelerate the adoption of cerium oxide nanomaterial-based wound care in clinical settings, there is a need to develop more degradable and absorbable green nanomaterials. In addition, there is a need to extend the functionality of nanomaterials by using gauze and hydrogels as carriers. This combination approach not only prevents acute toxicity caused by direct contact between nanomaterials and wounds, but also greatly expands the range of applications of nanomaterials in wound care, such as hemostasis, moisturization, and isolation from the external environment. And researchers have made several efforts to develop smart dressings with real-time monitoring and diagnostic capabilities, including detection and monitoring of wound-related bacteria, pH, temperature, movement, physiological signals, glucose, ROS, and uric acid. An example is the Bacterial Response Therapy Dressing. Early detection of wound infections will provide substantial benefits to physicians and patients by providing effective interventions. pH monitoring therapeutic dressing. pH is an important indicator of wound status as it is closely related to many physiologic processes including bacterial infection, angiogenesis and collagen formation. Temperature monitoring of therapeutic dressings. This is because it affects a range of chemical and enzymatic actions. Movement and physiologic signals monitor the therapeutic dressing. Because frequent movement at the wound site, compression and stretching of the wound can affect the growth of skin and muscle tissue, which can severely impact wound healing.

## 8 Conclusion

This article provides a review of the distinctive chemical structure and physicochemical properties of CeO_2_ NPs, as well as the recent progress in their application for wound healing. Furthermore, it discusses the growing significance of employing green synthesis methods for CeO_2_ NPs in wound healing applications. Effective and precise control over the surface chemical structure, particle size, and physicochemical properties of CeO_2_ NPs is necessary. Moreover, there have been recent advancements in utilizing CeO_2_ NPs in combination with other substances for wound healing. For example, precise control over the rate of drug release and dosage can lead to targeted therapy and enhanced efficacy. The feasibility and scientific validity of CeO_2_ NPs in clinical applications are still under investigation. In the future, it is expected that CeO_2_ will overcome additional challenges and thrive in interdisciplinary nanomedicine, thereby promoting its application in skin wound healing.

## References

[B1] Aalapati S. Ganapathy S. Manapuram S. Anumolu G. Prakya B. M. (2014). Toxicity and bio-accumulation of inhaled cerium oxide nanoparticles in CD1 mice. Nanotoxicology 8, 786–798. 10.3109/17435390.2013.829877 23914771

[B2] Abdal Dayem A. Hossain M. K. Lee S. B. Kim K. Saha S. K. Yang G.-M. (2017). The role of reactive oxygen species (ROS) in the biological activities of metallic nanoparticles. Int. J. Mol. Sci. 18, 120. 10.3390/ijms18010120 28075405 PMC5297754

[B3] Ahmad A. Javed M. S. Khan S. Almutairi T. M. Mohammed A. A. A. Luque R. (2023). Green synthesized Ag decoratedCeO_2_ nanoparticles: efficient photocatalysts and potential antibacterial agents. Chemosphere 310, 136841. 10.1016/j.chemosphere.2022.136841 36243088

[B4] Augustine R. Hasan A. Patan N. K. Dalvi Y. B. Varghese R. Antony A. (2020). Cerium oxide nanoparticle incorporated electrospun poly(3-hydroxybutyrate-Co-3-Hydroxyvalerate) membranes for diabetic wound healing applications. ACS Biomater. Sci. Eng. 6, 58–70. 10.1021/acsbiomaterials.8b01352 33463234

[B5] Augustine R. Zahid A. A. Hasan A. Dalvi Y. B. Jacob J. (2021). Cerium oxide nanoparticle-loaded gelatin methacryloyl hydrogel wound-healing patch with free radical scavenging activity. ACS Biomater. Sci. Eng. 7, 279–290. 10.1021/acsbiomaterials.0c01138 33320529

[B6] Babenko L. P. Zholobak N. M. Shcherbakov A. B. Voychuk S. I. Lazarenko L. M. Spivak M. Y. (2012). Antibacterial activity of cerium colloids against opportunistic microorganisms *in vitro* . Mikrobiol. Z 74, 54–62. 22830198

[B7] Bai Q. Han K. Dong K. Zheng C. Zhang Y. Long Q. (2020). Potential applications of nanomaterials and technology for diabetic wound healing. Int. J. Nanomedicine 15, 9717–9743. 10.2147/ijn.s276001 33299313 PMC7721306

[B8] Bai Y. Li Y. Li Y. Tian L. (2024). Advanced biological applications of cerium oxide nanozymes in disease related to oxidative damage. ACS Omega 9 (8), 8601–8614. 10.1021/acsomega.3c03661 38434816 PMC10905716

[B9] Baptista-Silva S. Borges S. Costa-Pinto A. R. Costa R. Amorim M. Dias J. R. (2021). *In situ* forming silk sericin-based hydrogel: a novel wound healing biomaterial. ACS Biomater. Sci. Eng. 7 (4), 1573–1586. 10.1021/acsbiomaterials.0c01745 33729761

[B10] Barbu A. Neamtu B. Zăhan M. Iancu G. M. Bacila C. Mireșan V. (2021). Current trends in advanced alginate-based wound dressings for chronic wounds. J. Pers. Med. 11 (9), 890. 10.3390/jpm11090890 34575668 PMC8471591

[B11] Betz C. Lenard A. Belting H.-G. Affolter M. (2016). Cell behaviors and dynamics during angiogenesis. Development 143, 2249–2260. 10.1242/dev.135616 27381223

[B12] Broughton G. Janis J. E. Attinger C. E. (2006). The basic science of wound healing. Plast. Reconstr. Surg. 117 (7 Suppl. l), 12S–34S. 10.1097/01.prs.0000225430.42531.c2 16799372

[B13] Burello E. Worth A. P. (2011). A theoretical framework for predicting the oxidative stress potential of oxide nanoparticles. Nanotoxicology 5, 228–235. 10.3109/17435390.2010.502980 21609138

[B14] Burgess J. L. Wyant W. A. Abdo Abujamra B. Kirsner R. S. Jozic I. (2021). Diabetic wound-healing science. Medicina 57 (10), 1072. 10.3390/medicina57101072 34684109 PMC8539411

[B15] Cañedo-Dorantes L. Cañedo-Ayala M. (2019). Skin acute wound healing: a comprehensive review. Int. J. Inflam. 2019, 10.1155/2019/3706315 PMC6582859 31275545

[B16] Carlander U. Moto T. P. Desalegn A. A. Yokel R. A. Johanson G. (2018). Physiologically based pharmacokinetic modeling of nanoceria systemic distribution in rats suggests dose- and route-dependent biokinetics. Int. J. Nanomedicine 13, 2631–2646. 10.2147/ijn.s157210 29750034 PMC5936012

[B17] Carvajal S. Perramón M. Casals G. Oró D. Ribera J. Morales-Ruiz M. (2019). Cerium oxide nanoparticles protect against oxidant injury and interfere with oxidative mediated kinase signaling in human-derived hepatocytes. Int. J. Mol. Sci. 20 (23), 5959. 10.3390/ijms20235959 31783479 PMC6928882

[B18] Castano C. E. O’Keefe M. J. Fahrenholtz W. G. (2015). Cerium-based oxide coatings. Curr. Opin. Solid State Mater Sci. 19 (2), 69–76. 10.1016/j.cossms.2014.11.005

[B19] Celardo I. De Nicola M. Mandoli C. Pedersen J. Z. Traversa E. Ghibelli L. (2011a). Ce³+ ions determine redox-dependent anti-apoptotic effect of cerium oxide nanoparticles. ACS Nano 5 (6), 4537–4549. 10.1021/nn200126a 21612305

[B20] Celardo I. De Nicola M. Mandoli C. Pedersen J. Z. Traversa E. Ghibelli L. (2011b). Ce^3+^ ions determine redox-dependent anti-apoptotic effect of cerium oxide nanoparticles. ACS Nano 5, 4537–4549. 10.1021/nn200126a 21612305

[B21] Charbgoo F. Ahmad M. B. Darroudi M. (2017a). Cerium oxide nanoparticles: green synthesis and biological applications. Int. J. Nanomedicine 12, 1401–1413. 10.2147/ijn.s124855 28260887 PMC5325136

[B22] Charbgoo F. Ahmad M. B. Darroudi M. (2017b). Cerium oxide nanoparticles: green synthesis and biological applications. Int. J. Nanomed. 12, 1401–1413. 10.2147/ijn.s124855 PMC5325136 28260887

[B23] Chen L. Deng H. Cui H. Fang J. Zuo Z. Deng J. (2017). Inflammatory responses and inflammation-associated diseases in organs. Oncotarget 9, 7204–7218. 10.18632/oncotarget.23208 29467962 PMC5805548

[B24] Chen Y. H. Rao Z. F. Liu Y. J. Liu X. S. Liu Y. F. Xu L. J. (2021). Multifunctional injectable hydrogel loaded with cerium-containing bioactive glass nanoparticles for diabetic wound healing. Biomolecules 11 (5), 702. 10.3390/biom11050702 34066859 PMC8151889

[B25] Cheng H. Shi Z. Yue K. Huang X. Xu Y. Gao C. (2021a). Sprayable hydrogel dressing accelerates wound healing with combined reactive oxygen species-scavenging and antibacterial abilitiesActa. Biomater 124, 219–232. 10.1016/j.actbio.2021.02.002 33556605

[B26] Cheng H. Shi Z. Yue K. Huang X. Xu Y. Gao C. (2021b). Sprayable hydrogel dressing accelerates wound healing with combined reactive oxygen species-scavenging and antibacterial abilities. Acta Biomater. 124, 219–232. 10.1016/j.actbio.2021.02.002 33556605

[B27] Cheng Y. Chang Y. Feng Y. Jian H. Wu X. Zheng R. (2019). Hierarchical acceleration of wound healing through intelligent nanosystem to promote multiple stages. ACS Appl. Mater Interfaces 11 (37), 33725–33733. 10.1021/acsami.9b13267 31449386

[B28] Chigurupati S. Mughal M. R. Okun E. Das S. Kumar A. McCaffery M. (2013). Effects of cerium oxide nanoparticles on the growth of keratinocytes, fibroblasts and vascular endothelial cells in cutaneous wound healing. Biomaterials 34 (9), 2194–2201. 10.1016/j.biomaterials.2012.11.061 23266256 PMC3552035

[B29] Clark A. Zhu A. Sun K. Petty H. R. (2011). Cerium oxide and platinum nanoparticles protect cells from oxidant-mediated apoptosis. J. Nanoparticle Res. 13, 5547. 10.1007/s11051-011-0544-3 PMC3203015 22039334

[B159] Comino-Sanz I. M. López-Franco M. D. Castro B. Pancorbo-Hidalgo P. L. (2021). The role of antioxidants on wound healing: a review of the current evidence. J. Clin. Med. 10 (16), 3558 34441854 10.3390/jcm10163558 PMC8397081

[B161] Wang Y. Chen S. Bao S. Yao L. Wen Z. Xu L. (2024). Deciphering the fibrotic process: mechanism of chronic radiation skin injury fibrosis. Front. immunol. 15, 1338922 38426100 10.3389/fimmu.2024.1338922 PMC10902513

[B30] Dahle J. T. Arai Y. (2015). Environmental geochemistry of cerium: applications and toxicology of cerium oxide nanoparticles. Int. J. Environ. Res. Public Health 12 (2), 1253–1278. 10.3390/ijerph120201253 25625406 PMC4344665

[B31] Damle M. A. Jakhade A. P. Chikate R. C. (2019). Modulating pro- and antioxidant activities of nanoengineered cerium dioxide nanoparticles against *Escherichia coli* . ACS Omega 4, 3761–3771. 10.1021/acsomega.8b03109

[B32] Darby I. A. Laverdet B. Bonté F. Desmoulière A. (2014). Fibroblasts and myofibroblasts in wound healing. Clin. Cosmet. Investig. Dermatol. 7, 301–311. 10.2147/CCID.S50046 PMC4226391 25395868

[B33] Darroudi M. Sarani M. Oskuee R. K. Zak A. K. Amiri M. S. (2014b). Nanoceria: gum mediated synthesis and *in vitro* viability assay. Ceram. Int. 40, 2863–2868. 10.1016/j.ceramint.2013.10.026

[B34] Darroudi M. Sarani M. Oskuee R. K. Zak A. K. Hosseini H. A. Gholami L. (2014a). Green synthesis and evaluation of metabolic activity of starch mediated nanoceria. Ceram. Int. 40, 2041–2045. 10.1016/j.ceramint.2013.07.116

[B35] Das M. Patil S. Bhargava N. Kang J. F. Riedel L. M. Seal S. (2007). Auto-catalytic ceria nanoparticles offer neuroprotection to adult rat spinal cord neurons. Biomaterials 28, 1918–1925. 10.1016/j.biomaterials.2006.11.036 17222903 PMC1913191

[B36] Das S. Chigurupati S. Dowding J. Munusamy P. Baer D. R. McGinnis J. F. (2014). Therapeutic potential of nanoceria in regenerative medicine. MRS Bull. 39 (11), 976–983. 10.1557/mrs.2014.221

[B37] Das S. Singh S. Dowding J. M. Oommen S. Kumar A. Sayle T. X. (2012b). The induction of angiogenesis by cerium oxide nanoparticles through the modulation of oxygen in intracellular environments. Biomaterials 33, 7746–7755. 10.1016/j.biomaterials.2012.07.019 22858004 PMC4590782

[B38] Das S. Singh S. Dowding J. M. Oommen S. Kumar A. Sayle T. X. (2012a). The induction of angiogenesis by cerium oxide nanoparticles through the modulation of oxygen in intracellular environments. Biomaterials 33 (31), 7746–7755. 10.1016/j.biomaterials.2012.07.019 22858004 PMC4590782

[B39] Datta A. Mishra S. Manna K. Saha K. D. Mukherjee S. Roy S. (2020). Pro-oxidant therapeutic activities of cerium oxide nanoparticles in colorectal carcinoma cells. ACS Omega 5, 9714–9723. 10.1021/acsomega.9b04006 32391458 PMC7203694

[B40] Del Turco S. Ciofani G. Cappello V. Parlanti P. Gemmi M. Caselli C. (2019). Effects of cerium oxide nanoparticles on hemostasis: coagulation, platelets, and vascular endothelial cells. J. Biomed. Mater Res. A 107 (7), 1551–1562. 10.1002/jbm.a.36669 30882978

[B41] de Oliveira R. C. Amoresi R. A. C. Marama N. L. Zaghete M. A. Chiquito A. J. Sambrano J. R. (2020). Influence of synthesis time on the morphology and properties of CeO_2_ nanoparticles. An experimental—theoretical study. Cryst. Growth Des. 20, 5031–5042. 10.1021/acs.cgd.0c00165

[B42] de Oliveira Gonzalez A. C. Costa T. F. de Araújo Andrade Z. Medrado A. R. A. P. (2016). Wound healing—a literature review. An. Bras. Dermatol. 91, 614–620. 10.1590/abd1806-4841.20164741 27828635 PMC5087220

[B43] Descamps B. Emanueli C. (2012). Vascular differentiation from embryonic stem cells: novel technologies and therapeutic promises. Vasc. Pharmacol. 56, 267–279. 10.1016/j.vph.2012.03.007 22504071

[B44] Dewberry L. C. Niemiec S. M. Hilton S. A. Louiselle A. E. Singh S. Sakthivel T. S. (2022). Cerium oxide nanoparticle conjugation to microRNA-146a mechanism of correction for impaired diabetic wound healing. Nanomedicine 40, 102483. 10.1016/j.nano.2021.102483 34748956 PMC9153729

[B45] Di Pietro L. A. (2013). Angiogenesis and scar formation in healing wounds. Curr. Opin. Rheumatol. 25, 87–91. 10.1097/bor.0b013e32835b13b6 23114588

[B46] Domínguez-Aragón A. Dominguez R. B. Zaragoza-Contreras E. A. (2021). Simultaneous detection of dihydroxybenzene isomers using electrochemically reduced graphene oxide-carboxylated carbon nanotubes/gold nanoparticles nanocomposite. Biosensors 11, 321. 10.3390/bios11090321 34562911 PMC8468658

[B47] Dutta D. Mukherjee R. Ghosh S. Patra M. Mukherjee M. Basu T. (2022). Cerium oxide nanoparticles as antioxidant or pro-oxidant agents. ACS Appl. Nano Mat. 5, 1690–1701. 10.1021/acsanm.1c04518

[B48] Ellis S. Lin E. J. Tartar D. (2018). Immunology of wound healing. Curr. Dermatol. Rep. 7, 350–358. 10.1007/s13671-018-0234-9 30524911 PMC6244748

[B49] Eriksson P. Tal A. A. Skallberg A. Brommesson C. Hu Z. Boyd R. D. (2018b). Cerium oxide nanoparticles with antioxidant capabilities and gadolinium integration for MRI contrast enhancement. Sci. Rep. 8, 6999. 10.1038/s41598-018-25390-z 29725117 PMC5934375

[B50] Eriksson P. Tal A. A. Skallberg A. (2018a). Cerium oxide nanoparticles with antioxidant capabilities and gadolinium integration for MRI contrast enhancement. Sci. Rep. 8 (1), 6999. 10.1038/s41598-018-25390-z 29725117 PMC5934375

[B51] Ezhilarasu H. Vishalli D. Dheen S. T. Bay B. H. Srinivasan D. K. (2020). Nanoparticle-based therapeutic approach for diabetic wound healing. Nanomaterials 10, 1234. 10.3390/nano10061234 32630377 PMC7353122

[B52] Fisichella M. Berenguer F. Steinmetz G. Auffan M. Rose J. Prat O. (2014). Toxicity evaluation of manufactured CeO2 nanoparticles before and after alteration: combined physicochemical and whole-genome expression analysis in Caco-2 cells. BMC Genomics 15 (1), 700. 10.1186/1471-2164-15-700 25145350 PMC4150968

[B53] Futosi K. Fodor S. Mócsai A. (2013). Neutrophil cell surface receptors and their intracellular signal transduction pathways. Int. Immunopharmacol. 17, 638–650. 10.1016/j.intimp.2013.06.034 23994464 PMC3827506

[B54] Gabbiani G. (2003). The myofibroblast in wound healing and fibrocontractive diseases. J. Pathol. 200, 500–503. 10.1002/path.1427 12845617

[B55] Gagnon J. Fromm K. (2015). Toxicity and protective effects of cerium oxide nanoparticles (nanoceria) depending on their preparation method, particle size, cell type, and exposure route. Eur. J. Inorg. Chem. 2015, 4510–4517. 10.1002/ejic.201500643

[B56] Gao L. Feng Q. Cui B. Mao Y. Zhao Z. Liu Z. (2023). Loading nanoceria improves extracellular vesicle membrane integrity and therapy to wounds in aged mice. ACS Biomater. Sci. Eng. 9 (2), 732–742. 10.1021/acsbiomaterials.2c01104 36642927

[B57] Ghahramani Z. Arabi A. M. Shafiee Afarani M. Mahdavian M. (2020). Solution combustion synthesis of cerium oxide nanoparticles as corrosion inhibitor. Int. J. Appl. Ceram. Technol. 17 (3), 1514–1521. 10.1111/ijac.13365

[B58] Gojova A. Lee J. T. Jung H. S. Guo B. Barakat A. I. Kennedy I. M. (2009). Effect of cerium oxide nanoparticles on inflammation in vascular endothelial cells. Inhal. Toxicol. 21 (Suppl. 1), 123–130. 10.1080/08958370902942582 19558244 PMC2859298

[B59] Gopinath K. Karthika V. Sundaravadivelan C. Gowri S. Arumugam A. (2015). Mycogenesis of cerium oxide nanoparticles using Aspergillus Niger culture filtrate and their applications for antibacterial and larvicidal activities. J. Nanostruct. Chem. 5, 295–303. 10.1007/s40097-015-0161-2

[B60] Guo S. DiPietro L. A. (2010). Factors affecting wound healing. J. Dent. Res. 89, 219–229. 10.1177/0022034509359125 20139336 PMC2903966

[B61] He S. Li Z. Wang L. Yao N. Wen H. Yuan H. (2024). A nanoenzyme-modified hydrogel targets macrophage reprogramming-angiogenesis crosstalk to boost diabetic wound repair. Bioact. Mater 35, 17–30. 10.1016/j.bioactmat.2024.01.005 38304915 PMC10831190

[B62] He S. Wu H. Huang J. Li Q. Huang Z. Wen H. (2023). 3-D tissue-engineered epidermis against human primary keratinocytes apoptosis via relieving mitochondrial oxidative stress in wound healing. J. Tissue Eng. 14, 20417314231163168. 10.1177/20417314231163168 37025157 PMC10071207

[B63] Hinz B. Gabbiani G. (2003). Cell-matrix and cell-cell contacts of myofibroblasts: role in connective tissue remodeling. Thromb. Haemost. 90 (6), 993–1002. 10.1160/TH03-05-0328 14652629

[B64] Hirst S. M. Karakoti A. S. Tyler R. D. Sriranganathan N. Seal S. Reilly C. M. (2009a). Anti-inflammatory properties of cerium oxide nanoparticles. Small 5 (24), 2848–2856. 10.1002/smll.200901048 19802857

[B65] Hirst S. M. Karakoti A. S. Tyler R. D. Sriranganathan N. Seal S. Reilly C. M. (2009b). Anti-inflammatory properties of cerium oxide nanoparticles. Small 5, 2848–2856. 10.1002/smll.200901048 19802857

[B66] Hosseini M. Amjadi I. Mohajeri M. Mozafari M. (2020). Sol-gel synthesis, physico-chemical and biological characterization of cerium oxide/polyallylamine nanoparticles. Polym. (Basel) 12 (7), 1444. 10.3390/polym12071444 PMC7407302 32605197

[B67] Hu Y. Du Y. Jiang H. Jiang G.-S. (2014). Cerium promotes bone marrow stromal cells migration and osteogenic differentiation via smad1/5/8 signaling pathway. Int. J. Clin. Exp. Pathol. 7 (8), 5369–5378. 25197425 PMC4152115

[B68] Huang J. Yu Y. Zhu J. Yu R. (2018). Oxygen adatoms and vacancies on the (110) surface of CeO_2_ . Sci. China Technol. Sci. 61 (1), 135–139. 10.1007/s11431-017-9154-9

[B69] Ighodaro O. M. Akinloye O. A. (2018). First line defence antioxidants-superoxide dismutase (SOD), catalase (CAT) and glutathione peroxidase (GPX): their fundamental role in the entire antioxidant defence grid. Alex. J. Med. 54, 287–293. 10.1016/j.ajme.2017.09.001

[B70] Ilina O. Friedl P. (2009). Mechanisms of collective cell migration at a glance. J. Cell Sci. 122, 3203–3208. 10.1242/jcs.036525 19726629

[B71] Ji Z. Wang X. Zhang H. Lin S. Meng H. Sun B. (2012). Designed synthesis of CeO2 nanorods and nanowires for studying toxicological effects of high aspect ratio nanomaterials. ACS Nano 6 (6), 5366–5380. 10.1021/nn3012114 22564147 PMC3651271

[B72] Johnson K. E. Wilgus T. A. (2014). Vascular endothelial growth factor and angiogenesis in the regulation of cutaneous wound repair. Adv. Wound Care 3, 647–661. 10.1089/wound.2013.0517 PMC4183920 25302139

[B73] Kaarniranta K. Pawlowska E. Szczepanska J. Jablkowska A. Blasiak J. (2019). Role of mitochondrial DNA damage in ROS-mediated pathogenesis of age-related macular degeneration (AMD). Int. J. Mol. Sci. 20, 2374. 10.3390/ijms20102374 31091656 PMC6566654

[B74] Kamalipooya S. Fahimirad S. Abtahi H. Golmohammadi M. Satari M. Dadashpour M. (2024). Diabetic wound healing function of PCL/cellulose acetate nanofiber engineered with chitosan/cerium oxide nanoparticles. Int. J. Pharm. 653, 123880. 10.1016/j.ijpharm.2024.123880 38350498

[B75] Kargar H. Ghazavi H. Darroudi M. (2015). Size-controlled and bio-directed synthesis of ceria nanopowders and their *in vitro* cytotoxicity effects. Ceram. Int. 41, 4123–4128. 10.1016/j.ceramint.2014.11.108

[B76] Kargozar S. Baino F. Hoseini S. J. Hamzehlou S. Darroudi M. Verdi J. (2018). Biomedical applications of nanoceria: new roles for an old player. Nanomedicine 13 (23), 3051–3069. 10.2217/nnm-2018-0189 30507347

[B77] Karimi A. Othman A. Andreescu S. (2016). Portable enzyme-paper biosensors based on redox-active CeO2 nanoparticles. Methods Enzymol. 571, 177–195. 10.1016/bs.mie.2016.03.006 27112400

[B78] Khurana A. Tekula S. Godugu C. (2018). Nanoceria suppresses multiple low doses of streptozotocin-induced Type 1 diabetes by inhibition of Nrf2/NF-κB pathway and reduction of apoptosis. Nanomedicine 13, 1905–1922. 10.2217/nnm-2018-0085 30152716

[B79] Koh T. J. DiPietro L. A. (2011). Inflammation and wound healing: the role of the macrophage. Expert Rev. Mol. Med. 13, e23. 10.1017/s1462399411001943 21740602 PMC3596046

[B80] Kyosseva S. V. Chen L. Seal S. McGinnis J. F. (2013). Nanoceria inhibit expression of genes associated with inflammation and angiogenesis in the retina of Vldlr null mice. Exp. Eye Res. 116, 63–74. 10.1016/j.exer.2013.08.003 23978600 PMC4263290

[B81] Li H. R. Xia P. Pan S. (2020).The advances of ceria nanoparticles for biomedical applications in orthopaedics.Int. J. Nanomed 15, 7199–7214. 10.2147/ijn.s270229 PMC7535115 33061376

[B82] Li J. Wang X. Yao Z. Yuan F. Liu H. Sun Z. (2023). NLRP3-Dependent crosstalk between pyroptotic macrophage and senescent cell orchestrates trauma-induced heterotopic ossification during aberrant wound healing. Adv. Sci. (Weinh) 10 (19), e2207383. 10.1002/advs.202207383 37204068 PMC10323626

[B83] Li J. H. Wen J. Li B. Li W. Qiao W. Shen J. (2018). Valence state manipulation of cerium oxide nanoparticles on a titanium surface for modulating cell fate and bone formation. Adv. Sci(Weinh) 5 (2), 1700678. 10.1002/advs.201700678 29610729 PMC5827567

[B84] Li S. Renick P. Senkowsky J. Nair A. Tang L. (2021). Diagnostics for wound infections. Adv. Wound Care (New Rochelle). 10 (6), 317–327. 10.1089/wound.2019.1103 32496977 PMC8082727

[B85] Li Y. Zhang W. Niu J. Chen Y. (2012). Mechanism of photogenerated reactive oxygen species and correlation with the antibacterial properties of engineered metal-oxide nanoparticles. ACS Nano 6, 5164–5173. 10.1021/nn300934k 22587225

[B86] Lin P. H. Sermersheim M. Li H. Lee P. H. U. Steinberg S. M. Ma J. (2017). Zinc in wound healing modulation. Nutrients 10 (1), 16. 10.3390/nu10010016 29295546 PMC5793244

[B87] Lindholm C. Searle R. (2016). Wound management for the 21st century: combining effectiveness and efficiency. Int. Wound J. 13, 5–15. 10.1111/iwj.12623 PMC7949725 27460943

[B88] Liu Z. Wang X. Xing Z. Xu P. Sun J. (2020). Nano-cerium oxide promotes proliferation of hepatoma cells and regulates mRNA expression of apoptosis-related genes bcl-2 and bax, as detected through real-time fluorescent quantitative polymerase chain reaction. J. Nanosci. Nanotechnol. 20, 7457–7463. 10.1166/jnn.2020.18718 32711615

[B89] Lu M. Zhang Y. Wang Y. Jiang M. Yao X. (2016). Insight into several factors that affect the conversion between antioxidant and oxidant activities of nanoceria. ACS Appl. Mat. Interfaces 8, 23580–23590. 10.1021/acsami.6b08219 27548073

[B90] Ludin A. Gur-Cohen S. Golan K. Kaufmann K. B. Itkin T. Medaglia C. (2014). Reactive oxygen species regulate hematopoietic stem cell self-renewal, migration and development, as well as their bone marrow microenvironment. Antioxid. Redox Signal. 21, 1605–1619. 10.1089/ars.2014.5941 24762207 PMC4175025

[B91] Luo J. Liu W. Xie Q. He J. Jiang L. (2023). Synthesis and characterisation of a novel poly(2-hydroxyethylmethacrylate)-chitosan hydrogels loaded cerium oxide nanocomposites dressing on cutaneous wound healing on nursing care of chronic wound. IET Nanobiotechnol 17 (4), 312–325. 10.1049/nbt2.12118 37312282 PMC10288362

[B92] Ma X. Cheng Y. Jian H. Feng Y. Chang Y. Zheng R. (2019). Hollow, rough, and nitric oxide-releasing cerium oxide nanoparticles for promoting multiple stages of wound healing. Adv. Healthc. Mater 8 (16), e1900256. 10.1002/adhm.201900256 31290270

[B93] Maccarone R. Tisi A. Passacantando M. Ciancaglini M. (2020). Ophthalmic applications of cerium oxide nanoparticles. J. Ocul. Pharmacol. Ther. 36 (6), 376–383. 10.1089/jop.2019.0105 31891528

[B94] Mackevica A. Hendriks L. Meili-Borovinskaya O. Baun A. Skjolding L. M. (2023). Effect of exposure concentration and growth conditions on the association of cerium oxide nanoparticles with green algae. Nanomater. (Basel) 13 (17), 2468. 10.3390/nano13172468 PMC10490049 37686976

[B95] Mai H. X. Sun L. D. Zhang Y. W. Si R. Feng W. Zhang H. P. (2005). Shape-selective synthesis and oxygen storage behavior of ceria nanopolyhedra, nanorods, and nanocubes. J. Phys. Chem. B 109 (51), 24380–24385. 10.1021/jp055584b 16375438

[B96] Mehta A. Scammon B. Shrake K. Bredikhin M. Gil D. Shekunova T. (2020). Nanoceria: metabolic interactions and delivery through PLGA-encapsulation. Mat. Sci. Eng. C 114, 111003. 10.1016/j.msec.2020.111003 32993995

[B97] Midwood K. S. Williams L. V. Schwarzbauer J. E. (2004). Tissue repair and the dynamics of the extracellular matrix. Int. J. Biochem. Cell Biol. 36, 1031–1037. 10.1016/j.biocel.2003.12.003 15094118

[B98] Nadeem M. Khan R. Afridi K. Nadhman A. Ullah S. Faisal S. (2020). Green synthesis of cerium oxide nanoparticles (CeO_2_ NPs) and their antimicrobial applications: a review. Int. J. Nanomedicine 15, 5951–5961. 10.2147/ijn.s255784 32848398 PMC7429212

[B99] Naganuma T. (2017). Shape design of cerium oxide nanoparticles for enhancement of enzyme mimetic activity in therapeutic applications. Nano Res. 10, 199–217. 10.1007/s12274-016-1278-4

[B100] Naidi S. N. Harunsani M. H. Tan A. L. Khan M. M. (2021). Green-synthesized CeO_2_ nanoparticles for photocatalytic, antimicrobial, antioxidant and cytotoxicity activities. J. Mater Chem. B 9 (28), 5599–5620. 10.1039/d1tb00248a 34161404

[B101] Nelson B. C. Johnson M. E. Walker M. L. Riley K. R. Sims C. M. (2016). Antioxidant cerium oxide nanoparticles in biology and medicine. Antioxidants 5, 15. 10.3390/antiox5020015 27196936 PMC4931536

[B102] Niemiec S. M. Louiselle A. E. Hilton S. A. Dewberry L. C. Zhang L. Azeltine M. (2020). Nanosilk increases the strength of diabetic skin and delivers CNP-miR146a to improve wound healing. Front. Immunol. 11, 590285. 10.3389/fimmu.2020.590285 33193424 PMC7662112

[B103] Nosrati H. Heydari M. Khodaei M. (2023). Cerium oxide nanoparticles: synthesis methods and applications in wound healing. Mater Today Bio 23, 100823. 10.1016/j.mtbio.2023.100823 PMC10622885 37928254

[B104] Nyoka M. Choonara Y. E. Kumar P. Kondiah P. P. D. Pillay V. (2020). Synthesis of cerium oxide nanoparticles using various methods: implications for biomedical applications. Nanomaterials 10, 242. 10.3390/nano10020242 32013189 PMC7075153

[B105] Özkan E. Cop P. Benfer F. Hofmann A. Votsmeier M. Guerra J. M. (2020). J. Phys. Chem. C 124 (16), 8736–8748.

[B106] Ozkan E. Cop P. Benfer F. Hofmann A. Votsmeier M. Guerra J. M. (2020). Rational synthesis concept for cerium oxide nanoparticles: on the impact of particle size on the oxygen storage capacity. J. Phys. Chem. C 124, 8736–8748. 10.1021/acs.jpcc.0c00010

[B107] Pandey S. Kumari S. Manohar Aeshala L. Singh S. (2024). Investigating temperature variability on antioxidative behavior of synthesized cerium oxide nanoparticle for potential biomedical application. J. Biomater. Appl. 38 (7), 866–874. 10.1177/08853282231226037 38173143

[B108] Park I.-S. Mahapatra C. Park J. S. Dashnyam K. Kim J.-W. Ahn J. C. (2020). Revascularization and limb salvage following critical limb ischemia by nanoceria-induced Ref-1/APE1-dependent angiogenesis. Biomaterials 242, 119919. 10.1016/j.biomaterials.2020.119919 32146371

[B109] Piipponen M. Li D. Landén N. X. (2020). The immune functions of keratinocytes in skin wound healing. Ijms 21, 8790. 10.3390/ijms21228790 33233704 PMC7699912

[B110] Plocon C. Evanghelidis A. Enculescu M. Isopencu G. Oprea O. Bacalum M. (2023). Development and characterization of electrospun composites built on polycaprolactone and cerium-containing phases. Int. J. Mol. Sci. 24 (18), 14201. 10.3390/ijms241814201 37762504 PMC10532413

[B111] Popova N. Andreeva V. V. Khohlov N. V. Popov A. Ivanov V. (2020). Fabrication of CeO_2_ nanoparticles embedded in polysaccharide hydrogel and their application in skin wound healing. Nanosyst. Phys. Chem. Math. 11, 99–109. 10.17586/2220-8054-2020-11-1-99-109

[B112] Purohit S. D. Singh H. Bhaskar R. Yadav I. Chou C.-F. Gupta M. K. (2020). Gelatin—alginate—cerium oxide nanocomposite scaffold for bone regeneration. Mat. Sci. Eng. C 116, 111111. 10.1016/j.msec.2020.111111 32806319

[B113] Raja I. S. Fathima N. N. (2018). Gelatin-cerium oxide nanocomposite for enhanced excisional wound healing. ACS Appl. Bio Mater 1 (2), 487–495. 10.1021/acsabm.8b00208 35016389

[B114] Ribera J. Rodríguez-Vita J. Cordoba B. Portolés I. Casals G. Casals E. (2019). PLoS One 14, e0218716. 10.1371/journal.pone.0218716 31233564 PMC6590813

[B115] Rivera-Briso A. L. Serrano-ArocaÁ (2018). Poly(3-Hydroxybutyrate-co-3-Hydroxyvalerate): enhancement strategies for advanced applications. Polym. (Basel) 10 (7), 732. 10.3390/polym10070732 PMC6403723 30960657

[B116] Růžička J. Dejmek J. Bolek L. Beneš J. Kuncová J. (2021). Hyperbaric oxygen influences chronic wound healing - a cellular level review. Physiol. Res. 70 (S3), S261–S273. 10.33549/physiolres.934822 35099246 PMC8884396

[B117] Rzigalinski B. A. Carfagna C. S. Ehrich M. (2017). Cerium oxide nanoparticles in neuroprotection and considerations for efficacy and safety. Wiley Interdiscip. Rev. Nanomed Nanobiotechnol 9 (4). 10.1002/wnan.1444 PMC5422143 27860449

[B118] Sadidi H. Hooshmand S. Ahmadabadi A. Javad Hosseini S. Baino F. Vatanpour M. (2020). Cerium oxide nanoparticles (nanoceria): hopes in soft tissue engineering. Molecules 25 (19), 4559. 10.3390/molecules25194559 33036163 PMC7583868

[B119] Saleh H. Nassar A. M. Noreldin A. E. Samak D. Elshony N. Wasef L. (2020). Chemo-protective potential of cerium oxide nanoparticles against fipronil-induced oxidative stress, apoptosis, inflammation and reproductive dysfunction in male white albino rats. Molecules 25, 3479. 10.3390/molecules25153479 32751827 PMC7435388

[B120] Sangha M. S. Deroide F. Meys R. (2024). Wound healing, scarring and management. Clin. Exp. Dermatol 49 (4), 325–336. 10.1093/ced/llad410 38001053

[B121] Sanmugam A. Sellappan L. K. Manoharan S. Rameshkumar A. Kumar R. S. Almansour A. I. (2024). Development of chitosan-based cerium and titanium oxide loaded polycaprolactone for cutaneous wound healing and antibacterial applications. Int. J. Biol. Macromol. 256 (Pt 1), 128458. 10.1016/j.ijbiomac.2023.128458 38016611

[B122] Seal S. Jeyaranjan A. Neal C. J. Kumar U. Sakthivel T. S. Sayle D. C. (2020). Engineered defects in cerium oxides: tuning chemical reactivity for biomedical, environmental. energy Appl. Nanoscale 12 (13), 6879–6899. 10.1039/d0nr01203c 32191231

[B123] Sener G. Hilton S. A. Osmond M. J. Zgheib C. Newsom J. P. Dewberry L. (2020). Injectable, self-healable zwitterionic cryogels with sustained microRNA - cerium oxide nanoparticle release promote accelerated wound healing. Acta Biomater. 101, 262–272. 10.1016/j.actbio.2019.11.014 31726250

[B124] Sharma G. Prema D. Venkataprasanna K. S. Prakash J. Sahabuddin S. Devanand Venkatasubbu G. (2020). Photo induced antibacterial activity of CeO_2_/GO against wound pathogens. Arab. J. Chem. 13, 7680–7694. 10.1016/j.arabjc.2020.09.004

[B125] Sharma M. Yadav S. Ganesh N. Srivastava M. M. Srivastava S. (2019). Biofabrication and characterization of flavonoid-loaded Ag, Au, Au-Ag bimetallic nanoparticles using seed extract of the plant Madhuca longifolia for the enhancement in wound healing bio-efficacy. Prog. Biomater. 8 (1), 51–63. 10.1007/s40204-019-0110-0 30790231 PMC6424993

[B126] Singh H. Purohit S. D. Bhaskar R. Yadav I. Bhushan S. Gupta M. K. (2021). Biomatrix from goat-waste in sponge/gel/powder form for tissue engineering and synergistic effect of nanoceria. Biomed. Mater 16 (2), 025008. 10.1088/1748-605x/abdb74 33440366

[B127] Singh K. R. Nayak V. Sarkar T. Singh R. P. (2020). Cerium oxide nanoparticles:properties,biosynthesis and biomedical application. RSC Adv. 10 (45), 27194–27214. 10.1039/d0ra04736h 35515804 PMC9055511

[B128] Singh S. Ly A. Das S. Sakthivel T. S. Barkam S. Seal S. (2018). Cerium oxide nanoparticles at the nano-bio interface: size-dependent cellular uptake. Artif. Cells Nanomed Biotechnol. 46 (Suppl. 3), S956–S963. 10.1080/21691401.2018.1521818 30314412

[B129] Soni S. Vats V. S. Kumar S. Dalela B. Mishra M. Meena R. S. (2018). Structural, optical and magnetic properties of Fe-doped CeO2 samples probed using X-ray photoelectron spectroscopy. J. Mat. Sci. Mat. Electron. 29, 10141–10153. 10.1007/s10854-018-9060-x

[B130] Sorg H. Tilkorn D. J. Hager S. Hauser J. Mirastschijski U. (2017). Skin wound healing: an update on the current knowledge and concepts. Eur. Surg. Res. 58 (1-2), 81–94. 10.1159/000454919 27974711

[B131] Stager M. A. Bardill J. Raichart A. Osmond M. Niemiec S. Zgheib C. (2022). Photopolymerized zwitterionic hydrogels with a sustained delivery of cerium oxide nanoparticle-miR146a conjugate accelerate diabetic wound healing. ACS Appl. Bio Mater 5 (3), 1092–1103. 10.1021/acsabm.1c01155 35167263

[B132] Stan D. Tanase C. Avram M. Apetrei R. Mincu N.-B. Mateescu A. L. (2021). Wound healing applications of creams and “smart” hydrogels. Exp. Dermatol. 30, 1218–1232. 10.1111/exd.14396 34009648 PMC8453519

[B133] Sun Z. Wu B. Ren Y. Wang Z. Zhao C. X. Hai M. (2021). Diverse particle carriers prepared by Co-precipitation and phase separation: formation and applications. Chempluschem 86 (1), 49–58. 10.1002/cplu.202000497 32894011

[B134] Thakur N. Manna P. Das J. (2019). Synthesis and biomedical applications of nanoceria, a redox active nanoparticle. J. Nanobiotechnol 17 (1), 84. 10.1186/s12951-019-0516-9 PMC6617741 31291944

[B135] Tracy L. E. Minasian R. A. Caterson E. J. (2016). Extracellular matrix and dermal fibroblast function in the healing wound. Adv. Wound Care 5, 119–136. 10.1089/wound.2014.0561 PMC4779293 26989578

[B136] Veith A. P. Henderson K. Spencer A. Sligar A. D. Baker A. B. (2019). Therapeutic strategies for enhancing angiogenesis in wound healing. Adv. Drug Deliv. Rev. 146, 97–125. 10.1016/j.addr.2018.09.010 30267742 PMC6435442

[B137] Velnar T. Bailey T. (2009). The wound healing process: an overview of the cellular and molecular mechanisms. J. Int. Med. Res. 37, 1528–1542. 10.1177/147323000903700531 19930861

[B138] Velnar T. Bailey T. Smrkolj V. (2009). The wound healing process: an overview of the cellular and molecular mechanisms. J. Int. Med. Red. 37, 1528–1542. 10.1177/147323000903700531 19930861

[B139] Walkey C. Das S. Seal S. Erlichman J. Heckman K. Ghibelli L. (2015). Catalytic properties and biomedical applications of cerium oxide nanoparticles. Environ. Sci. Nano 2 (1), 33–53. 10.1039/c4en00138a 26207185 PMC4508017

[B140] Wang L. Ai W. Zhai Y. Li H. Zhou K. Chen H. (2015). Effects of nano-CeO with different nanocrystal morphologies on cytotoxicity in HepG2 cells. Int. J. Environ. Res. Public Health 12 (9), 10806–10819. 10.3390/ijerph120910806 26404340 PMC4586644

[B141] Wang N. Liang H. Zen K. (2014). Molecular mechanisms that influence the macrophage M1-M2 polarization balance. Front. Immunol. 5, 1–9. 10.3389/fimmu.2014.00614 25506346 PMC4246889

[B142] Wang P. H. Huang B. S. Horng H. C. Yeh C. C. Chen Y. J. (2018). Wound healing. J. Chin. Med. Assoc. 81 (2), 94–101. 10.1016/j.jcma.2017.11.002 29169897

[B160] Wang Y. Yang L. Liu B. Liao S. Fu X. Zhou Y. (2023). Radiation skin injury care in radiotherapy for oncology: mechanisms, drug therapy and novel biomaterial application strategies. Adv. Therap. 6 (11), 2300024

[B143] Wei F. Neal C. J. Sakthivel T. S. Kean T. Seal S. Coathup M. J. (2021). Multi-functional cerium oxide nanoparticles regulate inflammation and enhance osteogenesis. Mat. Sci. Eng. C 124, 112041. 10.1016/j.msec.2021.112041 33947541

[B144] Wilkinson H. N. Hardman M. J. (2020a). Wound healing: cellular mechanisms and pathological outcomes: cellular mechanisms of wound repair. Open Biol. 10, 200223. 10.1098/rsob.200223 32993416 PMC7536089

[B145] Wilkinson H. N. Hardman M. J. (2020b). Wound healing: cellular mechanisms and pathological outcomes. Open Biol. 10 (9), 200223. 10.1098/rsob.200223 32993416 PMC7536089

[B146] Xu C. Qu X. (2014). Cerium oxide nanoparticle: a remarkably versatile rare earth nanomaterial for biological applications. NPG Asia Mat. 6 (3), e90. 10.1038/am.2013.88

[B147] Xu Y. Mofarah S. S. Mehmood R. Cazorla C. Koshy P. Sorrell C. C. (2021). Design strategies for ceria nanomaterials: untangling key mechanistic concepts. Mat. Horiz. 8, 102–123. 10.1039/d0mh00654h 34821292

[B148] Xue Y. Yang F. Wu L. Xia D. Liu Y. (2024). CeO2 nanoparticles to promote wound healing: a systematic review. Adv. Healthc. Mater 13 (6), e2302858. 10.1002/adhm.202302858 37947125

[B149] Yang B. Y. Zhou Z. Y. Liu S. Y. Shi M. J. Liu X. J. Cheng T. M. (2022). Porous Se@SiO2 nanoparticles enhance wound healing by ROS-PI3K/akt pathway in dermal fibroblasts and reduce scar formation. Front. Bioeng. Biotechnol. 10, 852482. 10.3389/fbioe.2022.852482 35387298 PMC8978548

[B150] Yildizbakan L. Iqbal N. Ganguly P. Kumi-Barimah E. Do T. Jones E. (2023). Fabrication and characterisation of the cytotoxic and antibacterial properties of chitosan-cerium oxide porous scaffolds. Antibiot. (Basel) 12 (6), 1004. 10.3390/antibiotics12061004 PMC10295205 37370323

[B151] Zeng Q. Qi X. Shi G. Zhang M. Haick H. (2022). Wound dressing: from nanomaterials to diagnostic dressings and healing evaluations. ACS Nano 16 (2), 1708–1733. 10.1021/acsnano.1c08411 35050565

[B152] Zgheib C. Hilton S. A. Dewberry L. C. Hodges M. M. Ghatak S. Xu J. (2019). Use of cerium oxide nanoparticles conjugated with MicroRNA-146a to correct the diabetic wound healing impairment. J. Am. Coll. Surg. 228 (1), 107–115. 10.1016/j.jamcollsurg.2018.09.017 30359833 PMC7846138

[B153] Zhang Z. Wang J. Luo Y. Li C. Sun Y. Wang K. (2023). A pH-responsive ZC-QPP hydrogel for synergistic antibacterial and antioxidant treatment to enhance wound healing. J. Mater Chem. B 11 (38), 9300–9310. 10.1039/d3tb01567j 37727911

[B154] Zhao R. Liang H. Clarke E. Jackson C. Xue M. (2016). Inflammation in chronic wounds. Int. J. Mol. Sci. 17, 2085. 10.3390/ijms17122085 27973441 PMC5187885

[B155] Zhao R. Zhao C. Wan Y. Majid M. Abbas S. Q. Wang Y. (2023). *In vitro* and *in vivo* evaluation of alginate hydrogel-based wound dressing loaded with green chemistry cerium oxide nanoparticles. Front. Chem. 11, 1298808. 10.3389/fchem.2023.1298808 38075491 PMC10701403

[B156] Zholobak N. Ivanov V. Shcherbakov A. (2016) Interaction of nanoceria with microorganisms. New York, NY, USA: Elsevier Inc., 419–450. 10.1016/b978-0-323-42864-4.00012-9

[B157] Zhu Z. Ding J. Qin M. Wang L. Jiang D. Zhao J. (2024). Enhanced ·OH-scavenging activity of Cu-CeOx nanozyme via resurrecting macrophage Nrf2 transcriptional activity facilitates diabetic wound healing. Adv. Healthc. Mater, 10.1002/adhm.202303229 38298062

[B158] Zuhrotun A. Oktaviani D. J. Hasanah A. N. (2023). Biosynthesis of gold and silver nanoparticles using phytochemical compounds. Molecules 28 (7), 3240. 10.3390/molecules28073240 37050004 PMC10096681

